# Identification and validation of paraptosis-related biomarkers in recurrent miscarriage

**DOI:** 10.3389/fimmu.2025.1656650

**Published:** 2025-11-05

**Authors:** Yunhui Wan, Fang Fang, Qiao Wang, Jia Xu, Pei Zhu, Ying Cui, Lili Hou, Huimin Wang, Xiaoyong Chen

**Affiliations:** ^1^ Department of Traditional Chinese Medicine, Jiangxi Maternal and Child Health Hospital, Nanchang, Jiangxi, China; ^2^ Jiangxi Key Laboratory of Reproductive Health, Nanchang, Jiangxi, China; ^3^ Key Research Unit of Female Reproduction with Integrated Chinese and Western Medicine of Jiangxi Province, Jiangxi Maternal and Child Health Hospital, Nanchang, Jiangxi, China; ^4^ Department of Traditional Chinese Medicine, Nanjing Maternity and Child Health Care Hospital, Nanjing, Jiangsu, China

**Keywords:** recurrent miscarriage, paraptosis, biomarker, single-cell RNA sequencing, experimental verification

## Abstract

**Background:**

Recurrent miscarriage (RM) is a pregnancy complication with growing evidence suggesting a role for paraptosis in its pathogenesis, though the underlying mechanisms remain unclear. This study investigated paraptosis-related genes (PRGs) as potential therapeutic targets.

**Methods:**

Transcriptome data for RM were obtained from public databases, while PRGs were sourced from existing literature. Biomarkers were identified through the intersection of differential expression analysis, weighted gene co-expression network analysis, machine learning algorithms and expression validation, followed by the construction and validation of a nomogram. Molecular mechanisms of the biomarkers were further explored through immune infiltration, enrichment analysis, and the construction of regulatory networks. Single-cell RNA sequencing (scRNA-seq) was performed for deeper insights into RM.

**Results:**

PCNPP3 and ELOA were selected as biomarkers related to paraptosis. A predictive nomogram was developed with strong accuracy. Enrichment analysis revealed that both PCNPP3 and ELOA were associated with E2F targets and the G2M checkpoint. In immune infiltration analysis, PCNPP3 exhibited a significant positive correlation with smooth muscle cells, while ELOA was notably associated with myocytes. Regulatory network analysis suggested that NEAT1 and NPPA-AS1 might modulate ELOA expression *via* hsa-miR-49-5p. ScRNA-seq analysis identified decidual natural killer (dNK) cells and macrophages as key cell types, with ELOA expression decreasing in dNK cells as their state changed, while in macrophages, expression followed a pattern of increase, decrease, and increase again.

**Conclusion:**

This study identified PCNPP3 and ELOA as biomarkers of RM and provides comprehensive insights into their molecular mechanisms, offering valuable perspectives for future RM research.

## 1 Introduction

Recurrent miscarriage (RM) is a significant reproductive health issue characterized by two or more consecutive pregnancy losses prior to the 20th week of gestation (1). Affecting approximately 1-5% of couples attempting conception, RM represents a common early pregnancy complication (2). Its multifactorial etiology involves genetic, anatomical, immunological, hormonal, and environmental factors (3). However, despite extensive research, 50-75% of RM cases remain idiopathic or unexplained, highlighting a critical gap in understanding the underlying mechanisms (4). Beyond its physical impact, RM often causes profound psychological distress and emotional trauma for individuals and couples, which can affect overall well-being and family dynamics (5).

Current diagnostic and therapeutic strategies for RM remain limited and often ineffective. While treatments such as pharmacological interventions, hormone therapy, and surgery are available, many patients show poor responses, underscoring the urgent need for the identification of reliable biomarkers and personalized treatment strategies (6). This further emphasizes the necessity of investigating the pathophysiological mechanisms underlying this disorder (7).

Non-apoptotic cell death, characterized by specific cell morphology and extensive cytoplasmic vacuolation during embryonic development or neuronal degeneration, is known as type III cell death or paraptosis (8). Paraptosis is a distinct form of programmed cell death, with cytoplasmic vacuolation being a hallmark feature, primarily resulting from the swelling of the endoplasmic reticulum and mitochondria (9). Under stress conditions such as oxidative stress or protein misfolding, the endoplasmic reticulum and mitochondria undergo significant swelling, forming vacuole-like structures within the cytoplasm (10). Paraptosis-related genes (PRGs) play a pivotal role in cancer treatment and regulation (11–13), and the paraptosis process has been described in various models (14, 15). However, its precise molecular mechanisms remain unclear (11). Previous studies suggest that paraptosis is regulated by various factors, including endoplasmic reticulum stress, proteasomal inhibition, reactive oxygen species production, and disturbances in cellular Ca^2+^ homeostasis (16). As a complex and dynamic process, paraptosis involves multiple factors, with the endoplasmic reticulum and mitochondria serving as critical organelles at the center of these processes (13). A unique aspect of the human reproductive cycle is the “spontaneous” deciduation of the endometrium in the absence of embryos, during which stromal cells undergo reticular stress and an unfolded protein response, leading to endoplasmic reticulum expansion and the production of immunomodulatory factors (17). Mitochondrial processes such as ATP synthesis, calcium ion storage, paraptosis induction, and ROS production significantly affect reproductive function (18, 19). PRGs in endometrial stromal cells, including endoplasmic reticulum stress markers such as CHOP and sXBP1, are upregulated in patients with RM, suggesting that endoplasmic reticulum dysfunction may play a pivotal role in this pathological condition (17). This form of cell death could impair the survival and function of placental cells, contributing to pregnancy failure. In animal models, endoplasmic reticulum stress has been shown to promote placental dysmorphogenesis, which is associated with pregnancy loss (20). Given these findings, it can be hypothesized that PRGs may also be involved in the pathogenesis of RM.

To further investigate this, the current study utilizes transcriptomic data related to RM to identify potential biomarkers linked to paraptosis. By constructing regulatory networks, performing enrichment analyses, and conducting immune infiltration assessments, the study aims to uncover the molecular mechanisms underlying the identified biomarkers. Additionally, single-cell RNA sequencing data will be used to examine the expression patterns of these biomarkers, identify key cell populations, and explore their differentiation pathways. This comprehensive approach seeks to deepen the understanding of RM and contribute to the development of targeted therapeutic strategies.

## 2 Materials and methods

### 2.1 Data acquisition

The GSE165004 (GPL1699), GSE111974 (GPL17077), and GSE214607 (GPL24676) datasets were sourced from the Gene Expression Omnibus (GEO) database (https://www.ncbi.nlm.nih.gov/geo/). The GSE165004 dataset served as the training set, originally comprising 72 samples. After excluding unexplained infertility samples, 24 endometrial tissue samples from individuals with RM and 24 corresponding control samples were selected for analysis. All RM samples were collected at the same stage of the menstrual cycle (LH + 7), ensuring the comparability of the samples. The RM group had a higher number of pregnancies but a lower number of deliveries, which was in line with the clinical characteristics of recurrent miscarriage. There was no significant difference in BMI between the two groups, ruling out the possibility of obesity as a confounding factor. The control group consisted of healthy women with a normal history of childbirth and no history of miscarriage, with regular menstrual cycles (25–35 days), and their ages were matched with those of the experimental group. Exclusion criteria for samples (applicable to both groups): Abnormal uterine anatomical structure, endocrine diseases (such as thyroid dysfunction, diabetes, etc.) (**Table 1**). The GSE111974 dataset, used as the validation set, contained 48 samples, including 24 endometrial samples from patients with RM and 24 control samples. GSE214607, a single-cell RNA sequencing dataset, included 16 samples, from which 3 decidual tissue samples from patients with RM and 5 endometrial tissue samples from controls were selected. The paraptosis-like genes (PRGs) adopted in this study were derived from systematic literature retrieval. The specific screening criteria were as follows: The literature was sourced from the PubMed database, with a time range up to August 2023. The screening criteria must explicitly mention genes related to paraptosis, which had been experimentally verified in human cells or tissues and are associated with cell death, endoplasmic reticulum stress, mitochondrial function, etc. Genes that had only been reported in mice or other models and have not been verified in humans were excluded. Then, a total of 66 PRGs were acquired from previous research (21) (**Supplementary Tables S1**).

**Table 1:** Baseline characteristics of the population.

Baseline category Group	Overall	Control	RM	P
n	48	24	24	
age	31.62 (± 4.24)	30.06 (± 2.39)	33.18 (± 5.10)	0.009**
bmi	22.81 (± 2.52)	22.39 (± 2.42)	23.22 (± 2.60)	0.259
gravidity	2.44 (± 1.43)	2.00 (± 1.06)	2.88 (± 1.62)	0.032*
parity	1.69 (1.52)	2.38 (1.66)	1.00 (± 0.98)	0.001**
abortion_history	1.00 (± 1.38)	0.00 (± 0.00)	2.00 (± 1.35)	<0.001***
menstrual_cycle	LH+7(100%)	LH+7(100%)	LH+7(100%)	NA

p < 0.05, **p < 0.01, ***p < 0.001, NA, Not involved.

### 2.2 Differential expression analysis

The Limma package (v 3.54.0) (22) was utilized to identify differentially expressed genes (DEGs) between the RM and control groups in the GSE165004 dataset (|log_2_ fold change (FC)| > 0.5 and p < 0.05). The ggplot2 package (v 3.4.4) (23) was employed to generate a volcano plot for the DEGs, marking the top 5 up- and down-regulated genes sorted by |log_2_FC|. A heatmap of the top 10 upregulated and downregulated DEGs sorted by |log_2_FC| was then created using ComplexHeatmap (v 2.14.0) (24).

### 2.3 Weighted gene co-expression network analysis

The WGCNA package (v 1.71) (25) was used to analyze gene modules associated with PRGs. Initially, the Wilcoxon test was applied to evaluate significant differences in the expression of PRGs between the RM and control groups in the GSE165004 dataset (p < 0.05). PRGs showing significant expression differences were selected for further analysis. Next, the GSVA package (v 1.46.0) (26) was utilized to calculate PRG scores, and the Wilcoxon test was again used to compare the differences between the RM and control cohorts in GSE165004 (p < 0.05). Afterward, all samples were clustered, and outliers were removed. To determine the optimal soft threshold for module construction, the scale-free topology model fitting index (R^2^) was set to 0.85. The optimal soft threshold was identified when R^2^ first exceeded this value and the average connectivity of the co-expression network approached zero, using the PickSoftThreshold function. An average connectivity approaching zero reduced redundant connections between modules, allowing for more specific co-expression within each module. The Dynamic Tree Cutting method was then applied to construct a scale-free network, with the minimum gene number per module set to 100, deepSplitG set to 4, and reassignThreshold set to 0.2, ensuring proper grouping of genes into distinct modules. Finally, Pearson’s correlation analysis was conducted with PRG scores as the trait to calculate correlation coefficients and p-values between the modules and the trait. Modules significantly correlated with the PRG scores (|correlation coefficient (cor)| > 0.3 and p < 0.05) were selected, and the genes within these modules were designated as key module genes. Finally, we conducted a sensitivity analysis via the WGCNA package (v 1.71) to verify the reliability of the results.

### 2.4 Protein-protein interaction relationships and functional evaluation of candidate genes

Candidate genes were identified by intersecting DEGs and key module genes using the ggvenn package (v 0.1.9) (27). To explore the biological functions of these candidate genes, clusterProfiler package (v 4.7.1.003) (28) was used to conduct Gene Ontology (GO) enrichment analysis. Although the False Discovery Rate (FDR) correction was applied, no terms remained statistically significant after this adjustment. Consequently, the results were selected based on a nominal p-value < 0.05 to identify potential biological trends. To construct the protein-protein interaction (PPI) network of candidate genes, this study used the Search Tool for Interacting Genes (STRING) database (https://string-db.org/). Since higher confidence thresholds (≥0.4, ≥0.7) yielded no or only very few interaction relationships, and to avoid missing potentially meaningful weak interactions, a threshold of 0.15 was finally selected. This choice was consistent with the feasible thresholds adopted in previous studies (29–31). The network was visualized using Cytoscape (v 3.9.1) (32).

### 2.5 Identification of biomarkers

**Figure 1:**
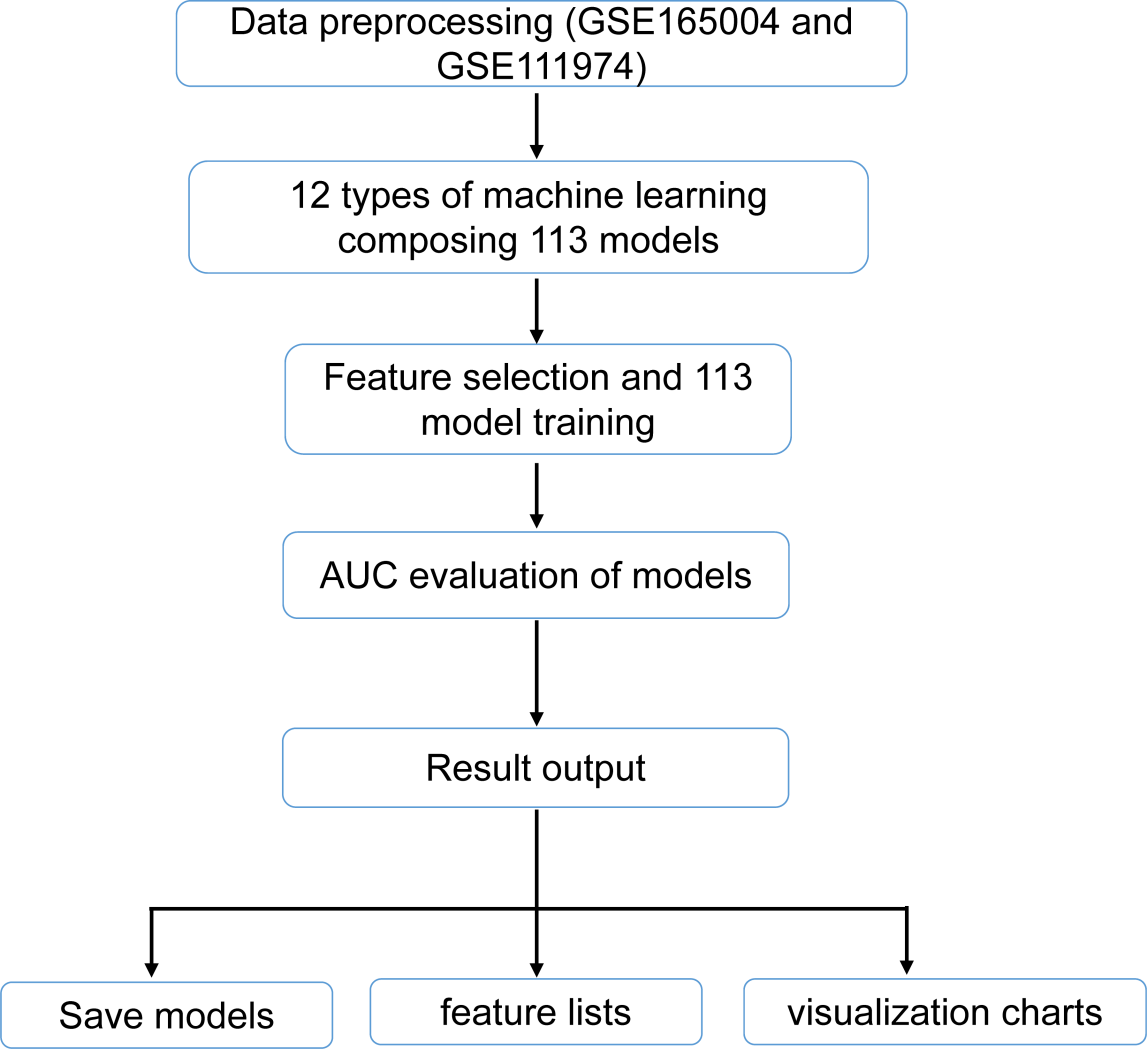
Flowchart of 113 machine learning methods.

For further refinement of candidate genes, 113 different combination models were constructed using 12 machine learning algorithms within a leave-one-out cross-validation (LOOCV) framework, based on the GSE165004 and GSE111974 datasets. The 12 algorithms used in this study included least absolute shrinkage and selection operator (LASSO), ridge regression, elastic net (elnet), stepwise generalized linear model (stepglm), support vector machine-recursive feature elimination (SVM-RFE), generalized linear model boosting (glmBoost), linear discriminant analysis (LDA), partial least squares regression regularized generalized linear model (plsRglm), random forest (RF), generalized boosted model (GBM), eXtreme gradient boosting (XGBoost), and NaiveBayes. Data preprocessing was initially carried out by retrieving datasets from GSE165004 and GSE111974, ensuring the inclusion of only the samples and expression data corresponding to the candidate genes across both datasets. Feature selection and model training followed, with feature subsets combined according to model configurations (e.g., “lasso+rf”) or derived from single methods (e.g., “lasso”) to enhance efficiency and consistency. The pROC package (v 1.18.0) (33) was employed to compute the area under the receiver operating characteristic (ROC) curve (AUC) for evaluating the classification performance of each model. Models were excluded based on their AUC values, and a heatmap was generated to visualize the AUC values of different model combinations in both the training and validation sets. **Figure 1** illustrates the analytical flowchart 113 models. The model with the highest AUC values (AUC ≥ 0.7) in both the training and validation sets was selected as the optimal model, and the genes within this model were designated as candidate biomarkers. The ROC curve of the optimal model was plotted to assess its performance.

The expression of candidate biomarkers in GSE165004 and GSE111974 was subsequently validated. Genes that exhibited significant expression differences between the RM and control groups (p < 0.05) and demonstrated consistent expression trends in both datasets were identified as biomarkers.

### 2.6 Constructed of nomogram

To assess the ability of these biomarkers to distinguish between RM and control groups, a nomogram based on their expression levels was constructed in GSE165004 using the rms package (v 6.5.0) (34). Calibration curves and ROC curves (AUC > 0.7) were generated using the rms and pROC packages (v 1.18.0) (33), respectively, to assess the accuracy of the nomogram in the GSE165004 and GSE111974.

### 2.7 Gene set enrichment analysis and gene set variation analysis

To investigate the biological functions and pathways associated with biomarkers in RM, GSEA was performed on the GSE165004 dataset. First, Spearman correlation coefficients between the biomarkers and all other genes were calculated using the psych package (v 2.2.9) (35). The correlation coefficients were then ranked from highest to lowest, and GSEA was conducted on the biomarkers using the clusterProfiler package (p < 0.05 and |normalized enrichment score (NES)| > 1). The reference gene set h.all.v2023.2.Hs.symbols.gmt was sourced from the Molecular Signatures Database (MisgDB, https://www.gsea-msigdb.org/gsea/msigdb).

Additionally, differences in enriched pathways between RM and control samples in GSE165004 were examined. The GSVA package was used to calculate the gene set scores for each sample, and the limma package assessed differences in gene expression (p.adj < 0.05). The pheatmap package (v 1.0.12) (36) was employed to generate heatmaps visualizing the top 10 pathways with the highest and lowest t-values, using the background set c2.all.v7.2.symbols.gmt.

### 2.8 Immune infiltration analysis

To explore the immune environment in RM, the xCell package (v 1.1.0) (37) was applied to assess the infiltration of 64 immune cell types (38) in RM and control groups within GSE165004. Immune cells exhibiting significant differences in infiltration (p < 0.05) were identified. The psych package was then used to evaluate the correlation between differential immune cells and biomarkers (|cor| > 0.3 and p < 0.05). In addition, the Single-sample Gene Set Enrichment Analysis (ssGSEA) algorithm of GSVA package (v 1.46.0) (26) was harnessed to determine the infiltration of 28 immune cells between RM and control groups in GSE165004, By comparing the infiltration of the 28 immune cells (p < 0.05), immune cells with significant differences were identified. Subsequently, the psych package was used to study the correlation between differential immune cells and biomarkers (with an absolute correlation value |cor| > 0.3 and p < 0.05).

### 2.9 Construction of regulatory networks

To analyze the regulatory relationships of biomarkers, upstream microRNAs (miRNAs) were predicted using the targetscan and miRDB databases within the multiMiR package (v 1.20.0) (39). The intersection of miRNAs from both databases identified key miRNAs. Additionally, the Starbase database was consulted to find upstream long non-coding RNAs (lncRNAs) for the identified key miRNAs. The regulatory network was then visualized using the ggraph package (v 2.1.0) (https://cloud.r-project.org/web/packages/ggraph/index.html).

### 2.10 Single-cell RNA sequence analysis

A series of single-cell analyses were performed to identify the key cells associated with biomarkers. The Seurat package (v 5.0.1) (40) was used to filter the GSE214607 dataset, applying the following criteria: 200 < nFeature_RNA count < 6000, nCount_RNA < 20, 000, and percent.mt < 10%. The LogNormalize function was then applied for data normalization, and high variability genes were identified using the FindVariableFeatures function. Principal component analysis (PCA) was conducted, and a scree plot was generated to determine the number of principal components (PCs) required for subsequent analyses. t-distributed stochastic neighbor embedding (T-SNE) was employed for cell clustering (resolution = 0.5). Based on clustering results and insights from single-cell RM literature (41), cell type annotation was performed, and the proportion of each cell type in different cohorts was displayed. Differential cell types were identified by comparing biomarker expression across all cell types (p < 0.05), with differential cells showing a higher proportion in the RM cohort selected as key cells. Next, the ReactomeGSA package (v 1.12.0) (42) was used to explore the biological functions associated with these differential cells. CellChat package (v 1.6.1) (43) was employed for cell-cell communication analysis. Subsequently, secondary clustering of the key cells was performed following the same procedure, and Monocle (v 2.26.0) (44) was utilized for pseudo-time analysis of the key cells.

### 2.11 Statistical analysis

Bioinformatics analyses were performed in R (v 4.2.2), using the Wilcoxon test for group comparisons, with p < 0.05 considered significant. The t-test was used for comparison of experimental data.

## 3 Results

### 3.1 There were 1, 467 DEGs and 259 key module genes ascertained

**Figure 2:**
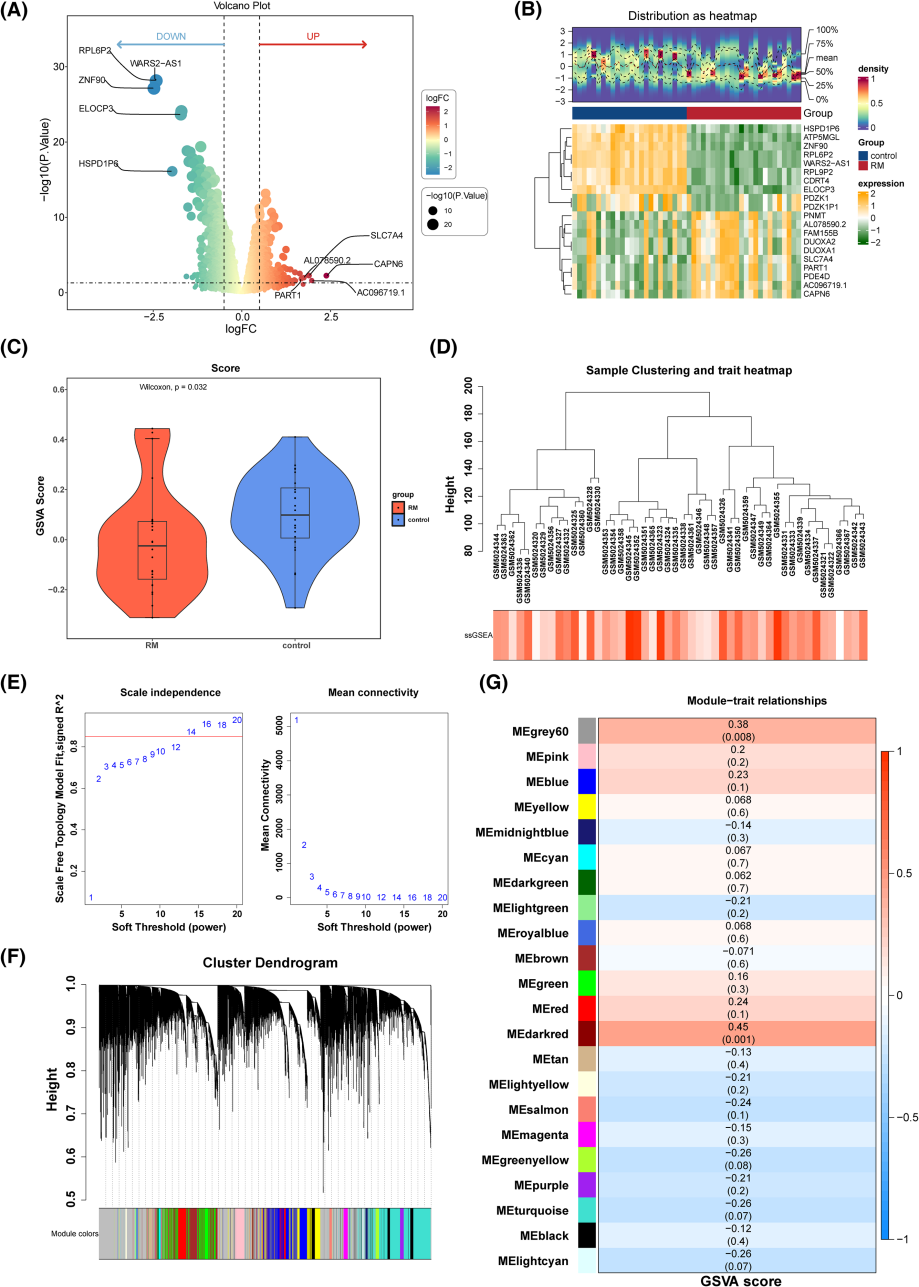
Recognition DEGs and key module genes. **(A)** Volcano plot of DEGs. **(B)** Heat map of the top 10 up-regulated genes and top 10 down-regulated genes. **(C)** PRGs GSVA score difference analysis violin chart between RM sample and control sample. **(D)** Sample clustering dendrogram of GSE165004. **(E)** The scale-free fit index for various softthresholding powers. **(F)** Clustering tree map of gene modules. **(G)** Heat map of correlation between genes in the module and PRGs scores.

In the GSE165004 dataset, 1, 467 DEGs were identified, including 648 up-regulated and 819 down-regulated genes in the RM cohort (**Figures 2A, B**). A gene co-expression network based on PRGs was subsequently constructed using WGCNA. Eighteen PRGs exhibited significantly different expression levels between the RM and control cohorts, with notable differences in their scores (p = 0.032) (**Figure 2C**). Hierarchical clustering analysis of all samples did not reveal any clear outliers (**Figure 2D**). An optimal soft threshold of 14 was determined, yielding an R^2^ of 0.8720 (**Figure 2E**). Hierarchical clustering further categorized the genes into 22 distinct co-expression modules (**Figure 2F**). The MEdarkred module (cor = 0.45, p = 0.001) and the MEgrey60 module (cor = 0.38, p = 0.008) were identified as key modules (**Figure 2G**), with the 259 genes within these modules defined as key module genes.The results of the sensitivity analysis showed that network construction was not sensitive to the selection of soft thresholds, and the identification of key module genes was not sensitive to changes in module size parameters. When the soft threshold was 14, the network not only maintains sufficient connectivity but also avoids overconnection, which conformed to the characteristics of a scale-free network. The above content enhances the reliability of the results (**Supplementary Figures 2A-C**).

### 3.2 The 30 candidate genes were identified

**Figure 3:**
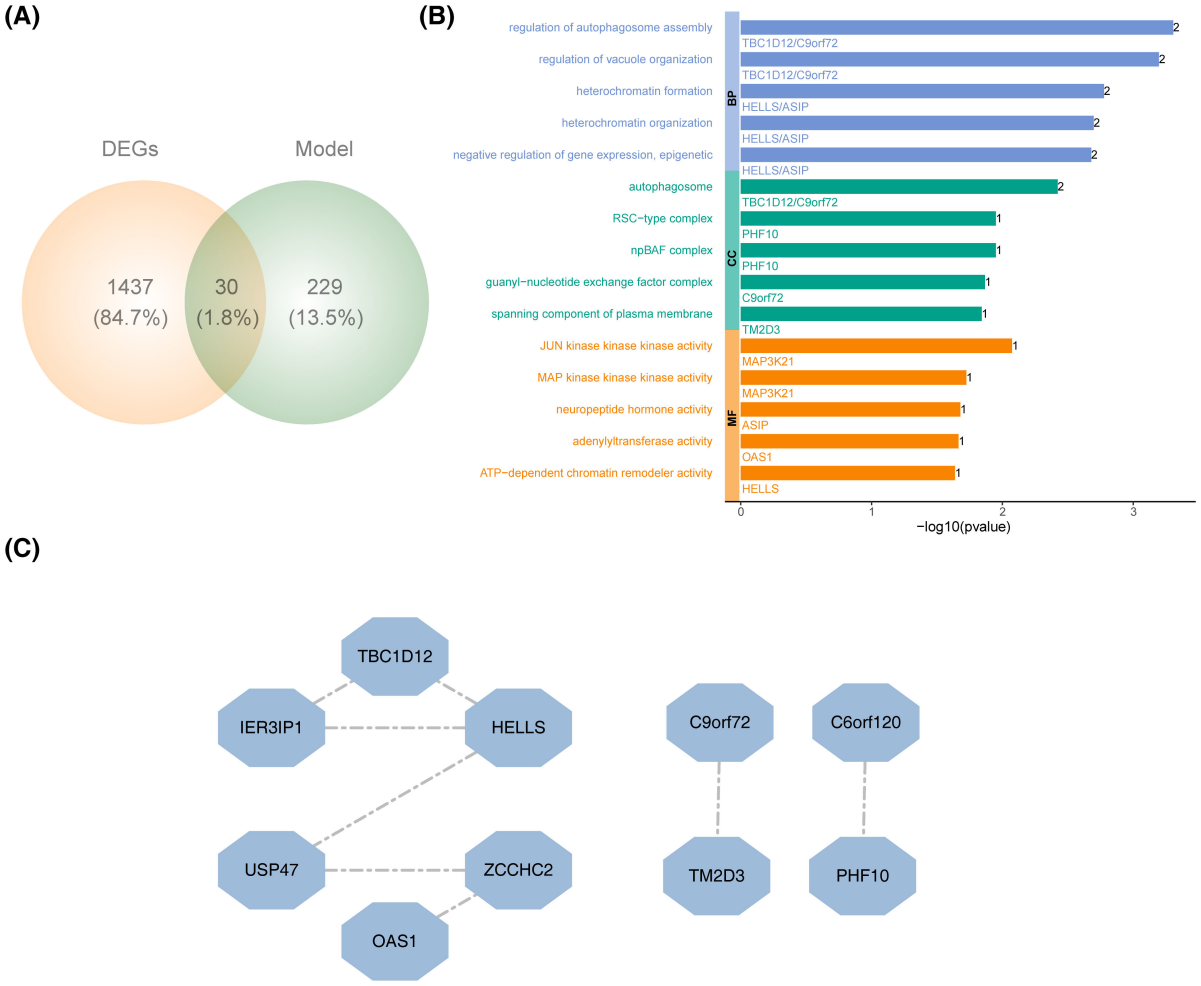
Recognition and functional annotation of candidate genes. **(A)** Venn diagram of DEGs and PRGs module genes. **(B)** GO enrichment analysis of candidate genes. (BP, biological process; CC, cellular component; MF, molecular function). **(C)** The PPI network construction of candidate genes.

By intersecting the DEGs with the key module genes, 30 candidate genes were identified (**Figure 3A**). GO analysis revealed that these candidate genes were enriched in 122 specific terms, including 14 cellular components, 8 molecular functions (MFs), and 100 biological processes (BPs). The top five enriched terms for cellular components, BPs, and MFs included pathways such as autophagosome, regulation of autophagosome assembly, and JUN kinase kinase kinase activity (**Figure 3B**). PPI analysis showed that only 10 of the candidate genes interacted with others, with HELLS displaying the strongest interaction potential (**Figure 3C**).

### 3.3 PCNPP3 and ELOA were considered as biomarkers

**Figure 4:**
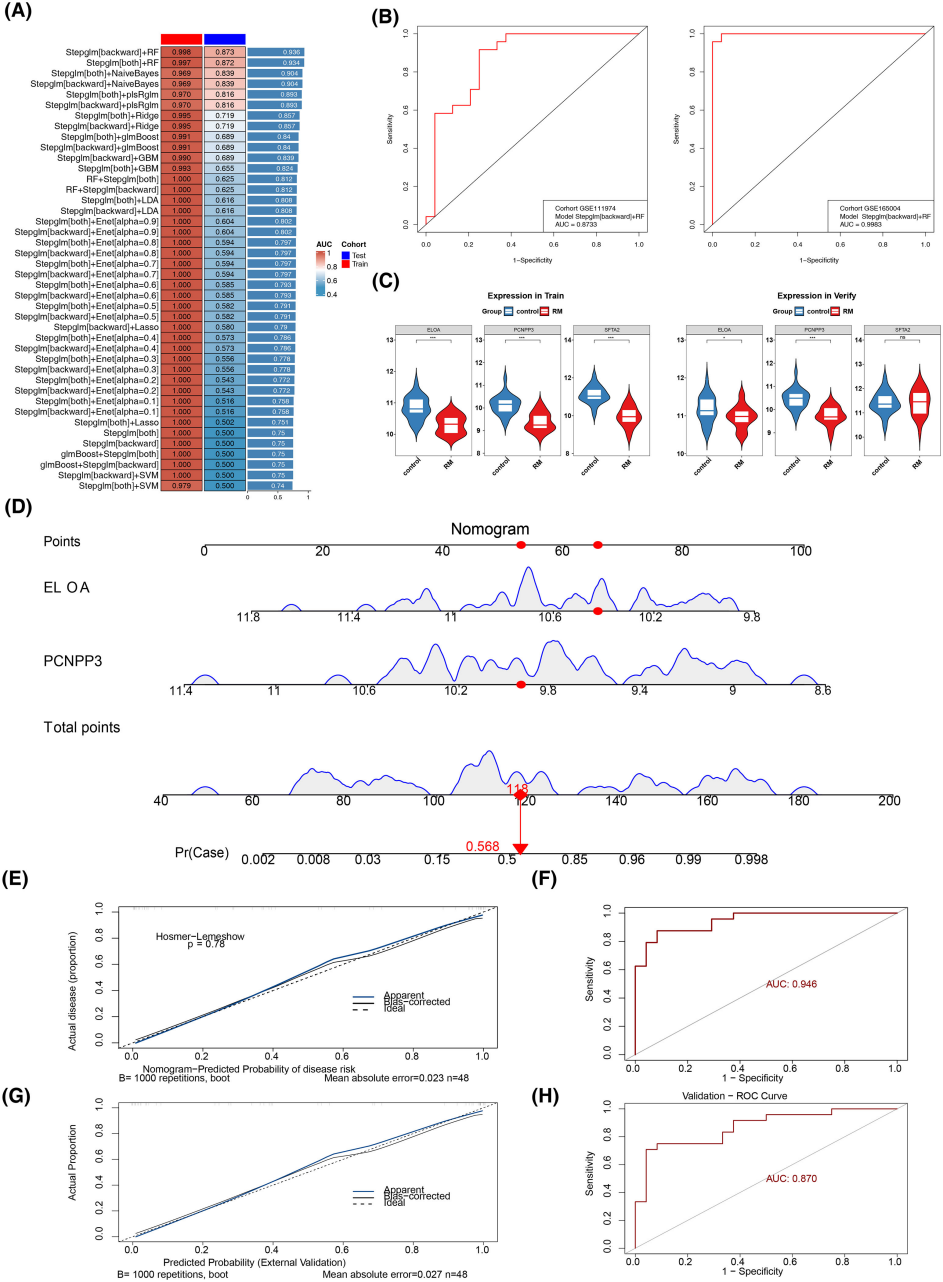
Biomarker Identification and Risk Assessment. **(A)** 113 joint models of the two datasets. **(B)** Distribution of the training set (left) and the validation set (right) on the ROC curve of the optimal model. **(C)** Box plots of the expression levels of candidate genes in the training set (left) and the validation set (right). **(D)** Construction of the nomogram **(E, G)** calibration curve. **(F, H)** ROC curve.

Subsequent results from 113 machine learning algorithm models indicated that the Stepglm[backward]+RF model had the best overall performance in both GSE165004 (AUC = 0.998) and GSE111974 (AUC = 0.873) (**Figures 4A, B**). This model was selected as the optimal one, with SFTA2, PCNPP3, and ELOA identified as candidate biomarkers. Further expression validation showed that in GSE165004, SFTA2, PCNPP3, and ELOA were significantly down-regulated in the RM cohort, while in GSE111974, only PCNPP3 and ELOA were significantly down-regulated in RM (**Figure 4C**). Thus, PCNPP3 and ELOA were considered key biomarkers for further analysis.

A nomogram was then constructed based on PCNPP3 and ELOA (**Figure 4D**). The calibration curve demonstrated a high degree of overlap between the nomogram curve and the reference line, with an AUC of 0.946 and 0.870, confirming that the nomogram had high diagnostic accuracy for RM (**Figures 4E–H**).

### 3.4 The biomarkers were associated with multiple pathways and immune cells

**Figure 5:**
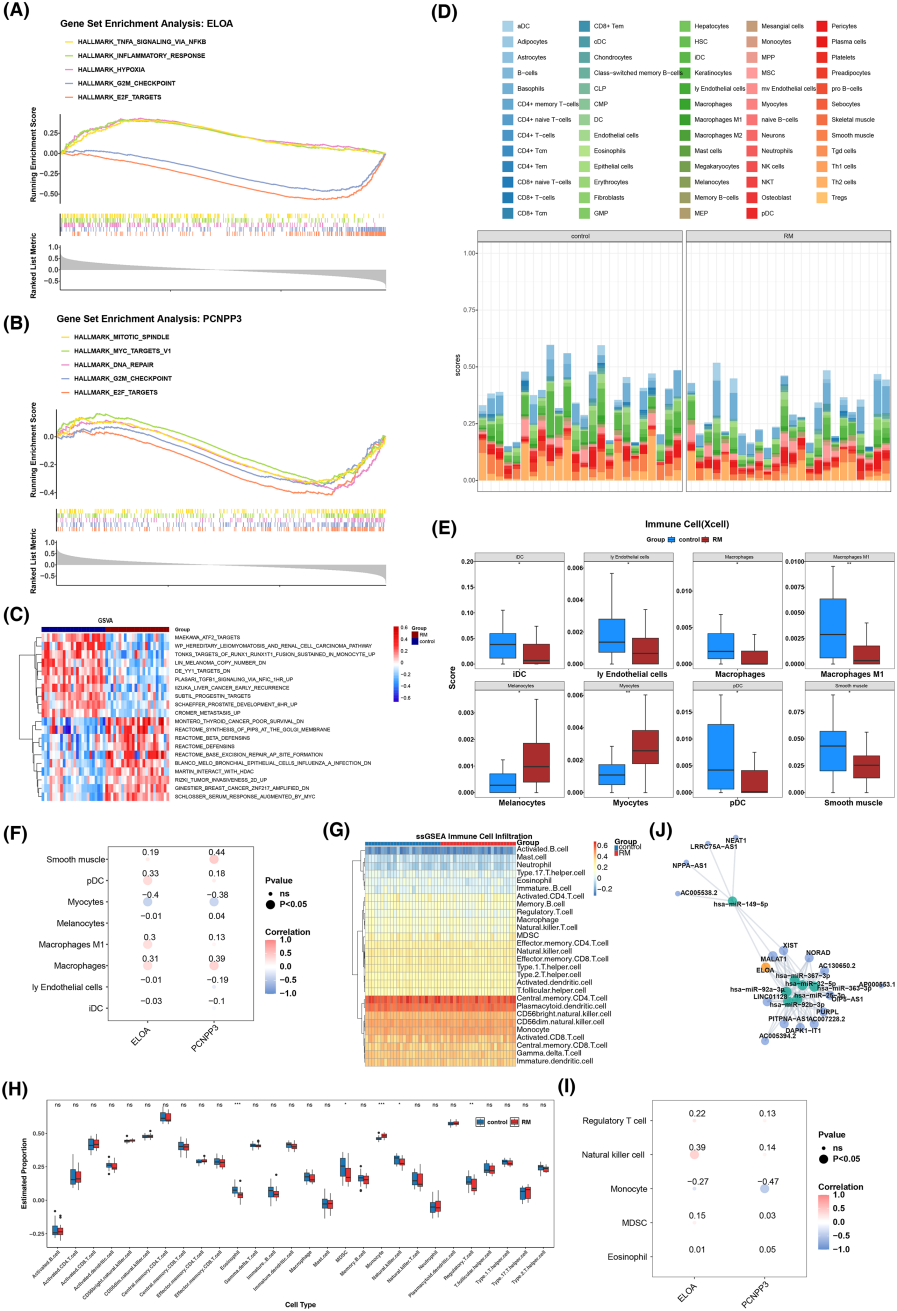
Systematic biological study of biomarkers. **(A)** GSEA enrichment analysis of ELOA. **(B)** GSEA enrichment analysis of PCNPP3. **(C)** GSVA analysis heat map between RM group and control group. **(D)** The infiltration and accumulation of 64 different types of immune cells in RM samples and control samples. **(E)** 8 differential immune cell types box diagram in the RM group and the control group.p < 0.05, **p < 0.01, **(F)** Pearman correlation analysis of key genes and differential immune cells. **(G)** The infiltration and accumulation of 28 different types of immune cells in RM samples and control samples. **(H)** 5 differential immune cell types box diagram in the RM group and the control group. p < 0.05, **p < 0.01, ***p < 0.001, ns, no significance **(I)** Pearman correlation analysis of key genes and differential immune cells. **(J)** Lncrna-mirna-key gene (mRNA) regulatory network based on ELOA, 7 key miRNAs and 16 lncRNAs.

GSEA results revealed that ELOA was significantly enriched in 30 pathways, while PCNPP3 was enriched in 19 pathways. Both genes were involved in the top five pathways, which included E2F targets and the G2M checkpoint (**Figures 5A, B**). Additionally, GSVA enrichment analysis identified 229 pathways, such as DE YY1 targets, ATF2 targets, and TONKS targets of RUNX1-RUNX1T1 fusion sustained in monocytes (**Figure 5C**).

The infiltration of 64 different immune cell types was assessed in RM and control samples (**Figure 5D**). Significant differences were observed in 8 immune cell types, with all but melanocytes and myocytes being significantly down-regulated in the RM cohort (**Figure 5E**). Spearman correlation analysis revealed that PCNPP3 was most strongly associated with smooth muscle cells (cor = 0.44, p < 0.05), while ELOA showed a significant negative correlation with myocytes (cor = -0.40, p < 0.05) (**Figure 5F**). The infiltration of 28 different types of immune cells in RM samples and control samples was shown in **Figure 5G**, with significant differences observed in 5 types of immune cells (**Figure 5H**). Following that, Spearman correlation analysis showed that PCNPP3 had the strongest negative with monocytes (cor = -0.47 and p < 0.05), while ELOA had the strongest significant positive linked to natural killer cells (cor = 0.39 and p < 0.05) (**Figure 5I**). Since xCell is mainly used to estimate the relative abundance of 64 types of immune and stromal cells, while ssGSEA is used to evaluate the activity of immune-related pathways and biological functions. These two methods characterize the immune status from different dimensions, so there may be certain differences in their results.

Using the Targetscan and miRDB databases, 40 and 22 miRNAs were predicted, respectively, and 7 key miRNAs were retained after intersection. The Starbase database was then used to predict 16 upstream lncRNAs for these miRNAs. A regulatory network was constructed around ELOA, the 7 key miRNAs, and 16 lncRNAs (**Figure 5J**).

### 3.5 The 14 differential cell types were annotated in GSE214607

**Figure 6:**
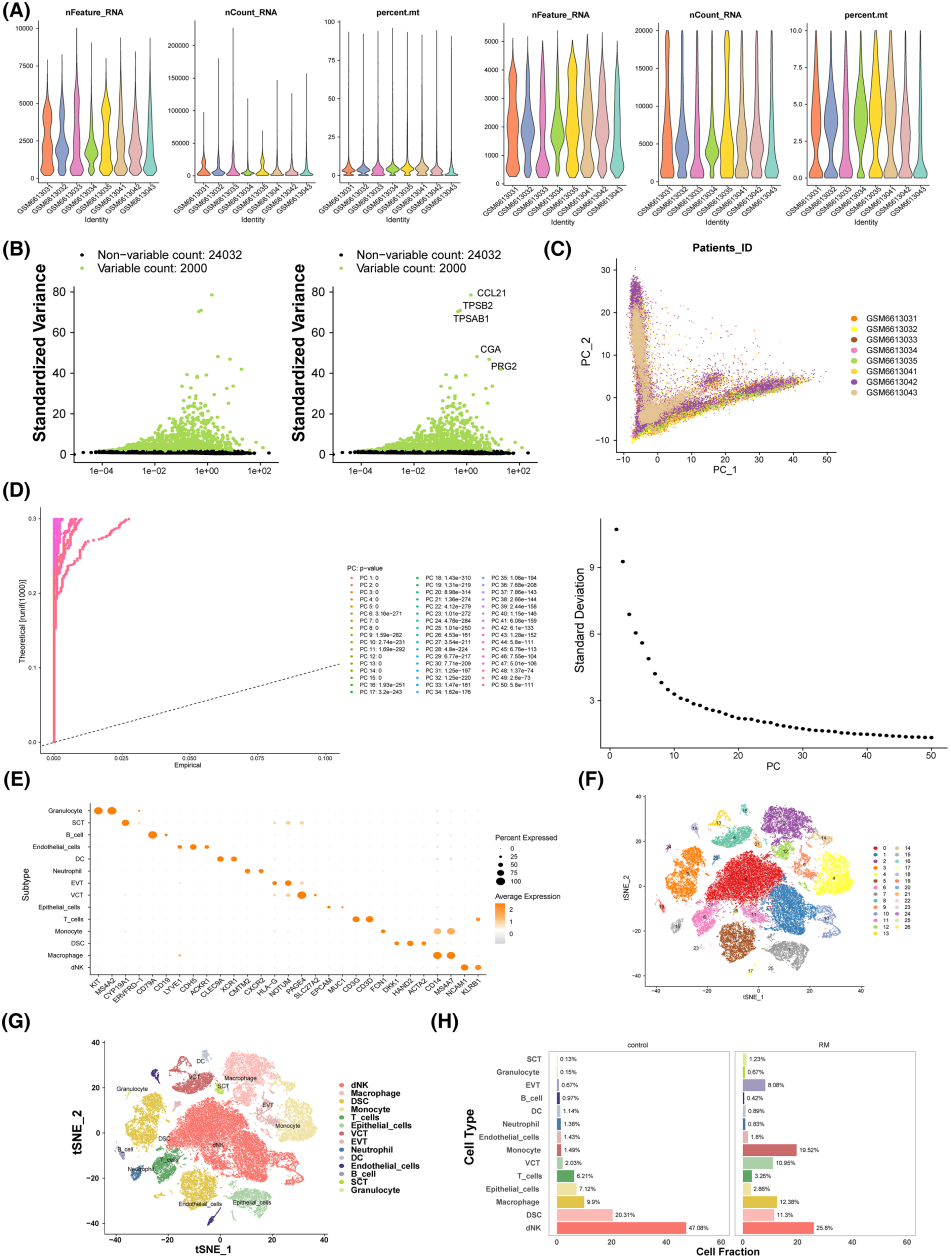
The scRNA-seq analysis of RM. **(A)** Violin chart of nFeature_RNA, nCount_RNA and percent.mt distribution before and after quality control. **(B)** High variant gene screening. **(C)** PCA analysis. **(D)** Linear dimension diagram and lithotripsy diagram. **(E)** t-SNE of the 27 cell cluster. **(F)** Relative expression of marker genes in cell clusters. **(G)** Annotated TSNE cluster diagram.Different colors represent different cell types. **(H)** Visualization of intergroup proportion in RM and control cohorts. The horizontal axis is the proportion of cells, and the vertical axis is the different cell types.

The distribution of gene count ranges, sequencing depth, and mitochondrial content ratios for all samples is shown in **Figure 6A**. Following rigorous quality control, 52, 077 cells and 26, 032 genes were retained for analysis. After data normalization, 2, 000 highly variable genes were identified, with the top 5 most variable genes highlighted, including CCL21 and TPSB2 (**Figure 6B**). PCA analysis revealed no clear boundaries between samples (**Figure 6C**), with data stabilization occurring after 30 PCs, which were selected for subsequent analysis (**Figure 6D**). t-SNE identified 27 distinct cell clusters (**Figure 6E**), which were annotated into 14 different cell types based on single-cell literature related to RM in the GSE214607 dataset. These cell types included granulocytes, SCT, B cells, endothelial cells, dendritic cells, neutrophils, extravillous trophoblasts (EVT), vascular tumor cells, epithelial cells, T cells, monocytes, dental stem cells, macrophages, and decidual natural killer cells (dNKs) (**Figures 6F, G**). In both RM and control cohorts, dNKs represented the largest proportion of cell types (**Figure 6H**).

### 3.6 The dNKs and macrophages were ascertained as key cells

**Figure 7:**
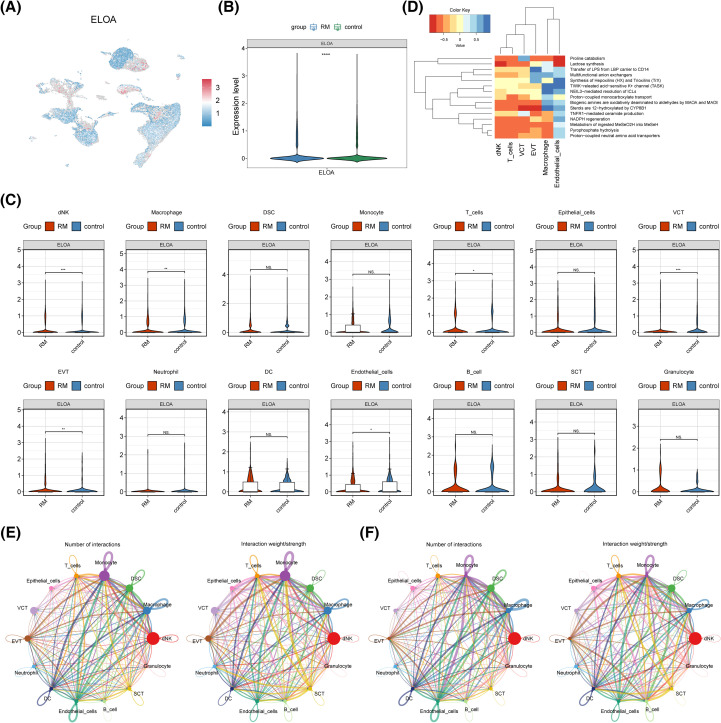
Key cell identification and cell communication. **(A)** TSNE diagram. Each dot represents a cell, and the closer it is to red, the higher the gene expression, and the closer it is to blue, the lower the gene expression. **(B)** ELOA expression in RM and control cohorts. **(C)** Expression of key genes between RM and control groups in all cells. ***p < 0.001, **p < 0.01, *p < 0.05, ns: *p*>0.05. **(D)** Heat map of cell functional enrichment. **(E, F)** Cell communication interaction diagram.

The expression of PCNPP3 and ELOA in GSE214607 revealed that ELOA was present in the single-cell dataset and exhibited notable differences between the RM and control cohorts (**Figures 7A, B**). Further analysis showed that ELOA expression was significantly distinct in dNK cells, macrophages, T cells, VCT, EVT, and endothelial cells (**Figure 7C**). Based on the proportion of these cells in the RM cohort, dNK cells and macrophages were defined as key cell types. Enrichment analysis of these key cells indicated their involvement in processes such as proline catabolism, NADPH regeneration, and lactose synthesis (**Figure 7D**).

Next, a communication analysis was conducted on the 14 cell types, revealing that dNK cells did not communicate with epithelial cells or VCT, and macrophages did not communicate with epithelial cells either (**Figures 7E, F**).

### 3.7 Development of key cells was correlated with the expression of key genes

**Figure 8:**
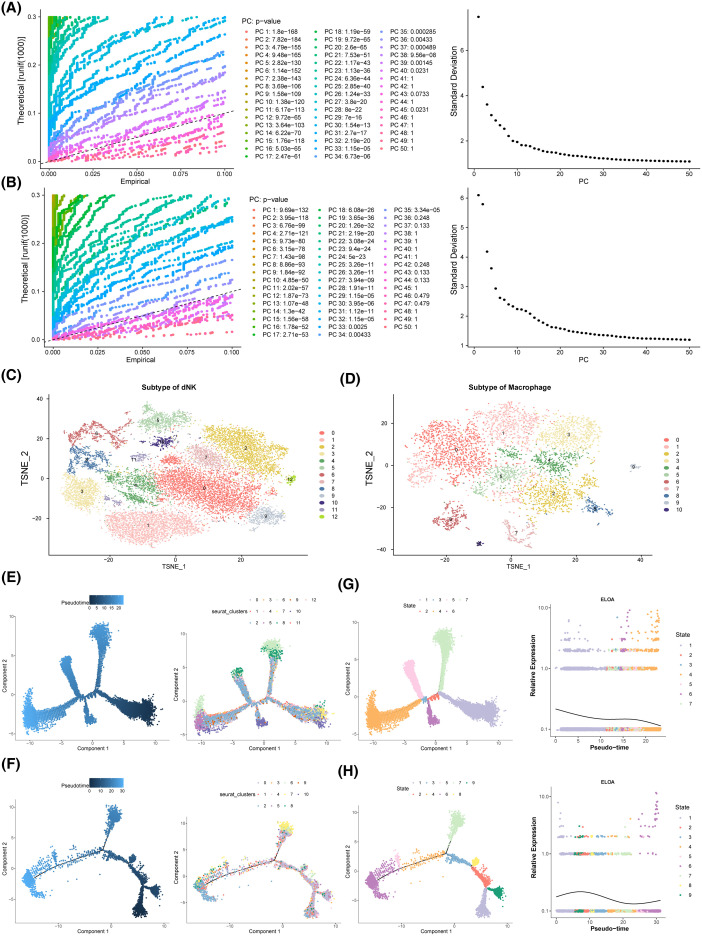
Pseudo-time analysis. **(A-D)** Analysis of key cell heterogeneity. **(A)** dNK cell dimension reduction analysis. **(B)** Macrophage dimension reduction analysis. **(C)** dNK cell cluster analysis. **(D)** Macrophage cluster analysis. **(E)** dNK pseudo-time series analysis. **(F)** Macrophage pseudo-time series analysis. **(G)** Expression of ELOA in dNK cells. **(H)** Expression of ELOA in Macrophage.

Dimensionality reduction and clustering were performed on dNK cells and macrophages. As shown in **Figures 8A, B**, both cell types stabilized at 30 PCs. dNK cells were further divided into 13 clusters (**Figure 8C**), while macrophages formed 11 clusters (**Figure 8D**). Pseudotime analysis of cellular trajectories revealed that dNK cells differentiated gradually from right to left, with cluster 2 present throughout the entire differentiation process, cluster 6 confined to the beginning and end of differentiation, and the entire process divided into 7 stages, with stage 3 being the shortest (**Figure 8E**). Macrophages differentiated from left to right, with cluster 5 present throughout the differentiation process, spanning 9 distinct stages, with stage 8 being the shortest (**Figure 8F**). ELOA expression in dNK cells decreased as differentiation progressed (**Figure 8G**), while in macrophages, it followed a pattern of increase, decrease, and then increase again (**Figure 8H**).

## 4 Discussion

RM is the most common clinical pathological pregnancy disorder, significantly impacting both the physical and mental health of patients, as well as their reproductive health (45). Its etiology is multifactorial, involving chromosomal abnormalities, autoimmune diseases, metabolic disorders, and more. However, the cause remains unknown in more than 50% of RM cases (3, 7). Although some biomarkers related to RM have been identified (46, 47), their clinical utility requires further validation. Additionally, many cases remain unexplained by known pathological mechanisms, highlighting the urgent need to discover new biomarkers and therapeutic options to improve RM diagnosis and treatment (48).

Recent studies (13, 16, 49) have focused on PRGs involved in cell death regulation, which play pivotal roles in paraptosis and autophagy, and may be closely linked to the pathological mechanisms of RM. PRGs have been shown to influence various biological processes, such as cell growth and death, offering new insights into the study of RM (15, 50). Study has shown that the natural compound tripterine can simultaneously induce paraptosis in cancer cells, accompanied by autophagy and apoptosis, confirming the concurrent occurrence of three programmed cell death patterns under the same stimulus (51). Additionally, research has indicated that endoplasmic reticulum stress and unfolded protein response can induce various cell death patterns, including apoptosis, autophagy, and ferroptosis. There are also common regulatory factors between paraptosis and various cell deaths, such as oxidative stress (52), indicating the association between paraptosis and other cell death patterns.

In this study, using 113 machine learning models, SFTA2, PCNPP3, and ELOA were identified as candidate biomarkers. Further expression validation retained PCNPP3 and ELOA as paraptosis-related biomarkers for subsequent analysis.

ELOA (Elongin A) is a transcriptional elongation factor that enhances the mRNA strand elongation rate of RNA polymerase II (53). Additionally, ELOA expression levels are closely associated with the development of various diseases. For instance, in tumor cells, high ELOA expression can promote cell proliferation and migration, thereby enhancing tumor aggressiveness and metastatic potential (12, 54). Additionally, ELOA may play a pivotal role in paraptosis by regulating intracellular signaling pathways, influencing cell sensitivity to anti-apoptotic signals (55). While the role of ELOA in RM has not been previously reported, this study found a significant downregulation of ELOA in RM decidual tissue (P < 0.0001). As an elongation factor of RNA polymerase II, ELOA is directly involved in the ubiquitination and degradation of Rpb1 (the largest subunit of RNA polymerase II) following DNA damage and plays a critical role in activating stress response genes (56). Moreover, ELOA has been confirmed as essential for early embryonic development. For example, experiments show that homozygous mutant Elongin A mice (Elongin A (-/-)) exhibit severely delayed embryonic development and die between days 10.5 and 12.5 of pregnancy. Mouse embryonic fibroblasts (MEF) derived from Elongin A (-/-) embryos show increased paraptosis and aging-like growth defects, along with the activation of p38 MAPK and p53 pathways. These findings suggest that ELOA may contribute to embryo loss through these mechanisms (57). These results provide novel perspectives for the early diagnosis and personalized treatment of RM.

In investigating the biological functions of the candidate biomarkers, the GSEA results highlighted the significant roles of ELOA and PCNPP3 in cell cycle regulation and cell proliferation. ELOA was significantly enriched in 30 pathways, while PCNPP3 was enriched in 19 pathways, with both genes involved in key pathways such as E2F targets and the G2M checkpoint. The E2F transcription factor family plays a critical role in regulating the cell cycle and promoting cell proliferation (58, 59). Additionally, E2F8 is particularly important in RM by regulating alpha-enolase 1 and its downstream signaling pathways. Specifically, E2F8 can positively regulate the expression of alpha-enolase 1 (60), which in turn activates the Wnt signaling pathway by inhibiting secreted Frizzled protein 1/4, thereby enhancing trophoblastic invasion—an essential process for maintaining a healthy pregnancy (60). The G2M checkpoint, a critical component of the cell cycle, monitors DNA damage and determines whether a cell can proceed to mitosis (61). During normal pregnancy, precise regulation of cell proliferation in both maternal and fetal tissues is necessary to ensure proper placental formation and function (60). Abnormal activation of the G2M checkpoint has been shown to lead to uncontrolled cell proliferation, disrupting embryo development and increasing the risk of miscarriage (62, 63). Genes associated with the G2M checkpoint, such as CDK1 and CCNB1, are upregulated in patients with RM, which may lead to adverse maternal responses to the embryo, potentially resulting in abortion (64, 65). Additionally, high G2M scores correlate with tumor mutation rates and immune cell infiltration, emphasizing the importance of this pathway in regulating the maternal immune environment (66). Several studies have also examined the interplay between the G2M pathway and other signaling pathways, such as MYC and E2F target genes (63, 67, 68). In summary, these pathways are integral to cell proliferation, paraptosis, and DNA repair, and their dysregulation may heighten the risk of RM. These pathways play a critical role in cell cycle regulation and may offer insight into the cellular dysfunctions linked to RM. The integration of pathway analysis with biomarker findings presents a multifaceted approach to understanding RM, suggesting that disruptions in cellular signaling and immune responses could be pivotal in its etiology. Therefore, ELOA may influence cell proliferation and genomic stability at the embryo-maternal interface through key cell cycle regulatory mechanisms (E2F/G2M-related pathways), contributing to the onset of RM. Targeting the E2F/G2M-related pathway could thus emerge as a potential therapeutic strategy for RM. Further functional experiments are needed to clarify its molecular targets and elucidate the upstream and downstream networks involved. PCNPP3 is a member of a necrotic protein gene family secreted by Phytophthora capsicum strains, classified as a pathogenic effector molecule. It primarily interacts with plant-specific receptors, initiating calcium ion influx, reactive oxygen species bursts, and allergic necrosis (69). To the best of our knowledge, the present study is the first to report the potential role of PCNPP3 in the human reproductive system, as it showed significant differential expression in the tissues of patients with RM (p < 0.001). While existing literature mainly describes the function of PCNPP3 in plant immune responses, such as hypersensitivity reactions (70), its potential role in mammalian systems has yet to be explored. Interestingly, some plant immune-related proteins share functional homologs in animal cells. For example, plant disease-resistant proteins, such as NLRs, have structural similarities with animal inflammasome components (71, 72), and plant cell death-related proteins, such as Metacaspases, function similarly to the paraptosis executive protein Caspase in animals (73). PCNPP3 may represent a new class of cross-species conserved proteins, with its core functional module potentially involved in cell fate regulation in both plant and animal systems. Based on “Immune-related protein functional homology between plants and animals”, it could be speculated that PCNPP3 might bind to the homologous conserved receptors at the maternal-fetal interface, mimicking the “receptor-ligand interaction” pattern in plants; it activaes abnormal calcium signals or ROS signals, ultimately triggering RM. Additionally, one of the important pathological mechanisms of RM was the insufficient invasive ability of trophoblast cells and the disorder of placental formation. The abnormal calcium/ROS signals activated by PCNPP3 may also directly inhibit the invasive ability of trophoblast cells (normal trophoblast cell invasion depends on precise calcium signal regulation), further hindering placental formation and ultimately increasing the risk of RM. However, this mechanism still requires more functional experiments for verification. Should the new function of PCNPP3 in mammals be confirmed, it could serve as a novel diagnostic marker for RM.

In an infiltration analysis of 64 immune cell types, significant differences were observed between the RM and control groups, with macrophages, melanocytes, smooth muscle cells, immature dendritic cells (iDC), lymphatic endothelial cells (ly Endothelial), plasmacytoid dendritic cells (pDC), M1 macrophages, and myocytes showing notable variations. All immune cells, except melanocytes and myocytes, were significantly down-regulated in the RM cohort. In a study by Ding et al. (74), macrophages inhibited TRAF6 expression at the post-transcriptional level through the transport of miR-146a-5p and miR-146b-5p, thus inhibiting epithelial-to-mesenchymal transition (EMT), migration, and invasion of trophoblast cells, contributing to the pathogenesis of recurrent spontaneous abortion (RSA). Other studies have similarly highlighted the important roles of macrophages, dendritic cells, and endothelial cells in regulating trophoblast activity in RM (41, 75–78). Subsequent Spearman correlation analysis revealed that PCNPP3 had the strongest significant correlation with smooth muscle cells, while ELOA exhibited the strongest negative correlation with muscle cells. These findings provide valuable insights into the immunological characteristics of RM and offer a reference point for future strategies aimed at improving reproductive outcomes by modulating the immune response.

This study constructed a regulatory network involving ELOA, key miRNAs, and upstream lncRNAs, offering a novel perspective for understanding the molecular mechanisms underlying RM. The network identified potential pathways through which lncRNAs, such as NEAT1 and NPPA-AS1, might regulate ELOA expression by targeting hsa-miR-49-5p. NEAT1 has been shown to be associated with the development of various tumors (79) and plays a role in pulmonary fibrosis (80). In pregnancy-related diseases, the regulatory function of NEAT1 has been increasingly recognized. For instance, in preeclampsia, NEAT1 can inhibit trophoblast cell proliferation (81). Previous studies have reported that miR-49-5p in placental trophoblast cells regulates cell survival by targeting paraptosis-related genes, suggesting its potential involvement in maternal-fetal interface immune tolerance (74). Abnormal expression of NEAT1 may lead to reduced ELOA expression by sponging miR-49-5p, thereby impacting decidual cell proliferation and the embryonic developmental microenvironment (82). The discovery of this “lncRNA-miRNA-mRNA” regulatory axis expands our understanding of RM’s molecular mechanisms, shifting the focus from a single gene to a complex network level and highlighting the central role of non-coding RNAs in regulating the maternal-fetal interface.

dNK cells are the most abundant immune cell population at the maternal-fetal interface. They promote the remodeling of spiral arterioles in the decidua by facilitating the invasion of EVT cells and interacting with them during early pregnancy. As pregnancy progresses, dNK cells help clear decidualized cells, thereby maintaining endometrial balance and ensuring a normal physiological state post-implantation (83, 84). Zhang et al. (85) showed that dNK cells promote decidualization by secreting interleukin 25. However, in miscarriage patients, the number of dNK cells is reduced, accompanied by elevated TNF-α levels, which inhibit decidualization by decreasing the expression of decidualization markers such as PRL and IGFBP-1 (86). Moreover, CD39 and CD73 levels were significantly lower in the tissues of patients with unexplained RM compared to those in normal gestation, leading to increased toxicity and decreased paraptosis of dNK cells (87). Therefore, changes in the function or number of dNK cells may disrupt decidualization, ultimately contributing to RM. Macrophages are key immune cells in the decidual tissue, playing an essential role in embryo implantation and pregnancy maintenance (88). Both M1 and M2 macrophages participate in angiogenesis and immune suppression at the maternal-fetal interface (89). Abnormal polarization of macrophages is closely linked to unexplained RSA (90). In the present study, single-cell RNA sequencing revealed that ELOA was expressed in the single-cell dataset and showed significant differences between the RM and control groups. Further pseudotime analysis indicated that ELOA expression in dNK cells gradually decreased as the cellular state changed, whereas in macrophages, ELOA expression exhibited a dynamic trend of initially increasing, then decreasing, and increasing again. These findings highlight the heterogeneity within immune cell populations, particularly in dNK cells and macrophages, emphasizing their distinct roles during pregnancy. Fluctuations in ELOA expression in dNK cells and its dynamic regulation in macrophages suggest that these immune cells may play pivotal roles in modulating the uterine environment during early pregnancy.

This study highlights the multi-dimensional correlations among genes, immune cells, and regulatory networks, thereby enhancing the understanding of the immune mechanisms underlying diseases and offering potential diagnostic markers, therapeutic targets, and individualized treatment strategies for clinical application. The expression levels of ELOA and PCNPP3 are significantly associated with the infiltration of various immune cells, such as smooth muscle cells and myocytes, suggesting that these two genes may contribute to the development and progression of diseases like RM by regulating the immune microenvironment (91). For instance, the negative correlation between ELOA and myocytes may indicate its involvement in the pathological process by influencing the immune homeostasis or cell function of muscle tissue (92). Both ELOA and PCNPP3 are significantly downregulated in RM samples and are linked to the differentiation trajectories of key immune cells, suggesting that their expression levels could serve as diagnostic or prognostic markers for RM. For example, assessing ELOA expression in decidual tissue may aid in evaluating the risk of pregnancy failure or distinguishing between normal and pathological pregnancies (93). Abnormal proportions of dNK cells and macrophages in the RM group, such as changes in dNK cell proportions, could serve as early warning indicators of immune imbalance. Monitoring these proportions using single-cell sequencing or flow cytometry may provide a foundation for individualized clinical treatment (94).

Although the research results are encouraging, this study still has some limitations. Firstly, the research results are based on bioinformatics analysis and lack *in vivo* and *in vitro* experiments for validation to confirm the biological functions of PCNPP3 and ELOA in RM. Secondly, the analysis is limited by the size of the existing cohort and the scarcity of single-cell datasets, which may affect the generalizability of the results and the in-depth understanding of the cellular-level mechanisms. Future research can proceed in the following directions: Firstly, it is necessary to obtain larger-scale, multi-center RM-related datasets, and focus on the external validation of the nomogram model and the expression stability assessment of biomarkers in independent cohorts; Secondly, single-cell sequencing technology should be used to deeply analyze the endometrial samples of RM patients and controls, to clarify the expression patterns and key cell characteristics of paraptosis-related biomarkers in specific cells; Moreover, the sample size of clinical cohorts should be further expanded and rigorous statistical analysis should be adopted to reduce the interference of confounding factors, and animal models and other *in vivo* experiments should be used to verify the functional mechanism of PCNPP3 and ELOA in RM, providing a more solid theoretical basis and practical guidance for the clinical diagnosis, treatment and prognosis assessment of RM.

In conclusion, PCNPP3 and ELOA have been identified as paraptosis-related biomarkers for RM for the first time. This discovery opens new avenues for studying their specific roles in the paraptosis processes of RM cells and presents new targets and research directions for the treatment of RM.

## Data Availability

The datasets ANALYZED for this study can be found in the[Gene Expression Omnibus (GEO) database] [http://www.ncbi.nlm.nih.gov/geo/, GSE165004, GSE111974, and GSE214607 (GPL24676)]. The original contributions presented in the study are included in the article/**Supplementary Material**. Further inquiries can be directed to the corresponding author.

## References

[1] Bhardwaj C Srivastava P. Identification of hub genes in placental dysfunction and recurrent pregnancy loss through transcriptome data mining: A meta-analysis. Taiwan J Obstet Gynecol. (2024) 63:297–306. doi: 10.1016/j.tjog.2024.01.035, PMID: 38802191

[2] Nikitina TV Sazhenova EA Zhigalina DI Tolmacheva EN Sukhanova NN Lebedev IN. Karyotype evaluation of repeated abortions in primary and secondary recurrent pregnancy loss. J Assist Reprod Genet. (2020) 37:517–25. doi: 10.1007/s10815-020-01703-y, PMID: 32009222 PMC7125272

[3] Wartena R Matjila M. Polycystic ovary syndrome and recurrent pregnancy loss, a review of literature. Front Endocrinol (Lausanne). (2023) 14:1183060. doi: 10.3389/fendo.2023.1183060, PMID: 38027110 PMC10643146

[4] Turesheva A Aimagambetova G Ukybassova T Marat A Kanabekova P Kaldygulova L. Recurrent pregnancy loss etiology, risk factors, diagnosis, and management. Fresh Look into Full Box J Clin Med. (2023) 12:4074. doi: 10.3390/jcm12124074, PMID: 37373766 PMC10298962

[5] Shi Y Tan D Hao B Zhang X Geng W Wang Y. Efficacy of intravenous immunoglobulin in the treatment of recurrent spontaneous abortion: A systematic review and meta-analysis. Am J Reprod Immunol. (2022) 88:e13615. doi: 10.1111/aji.13615, PMID: 36029201 PMC9787751

[6] Dimitriadis E Menkhorst E Saito S Kutteh WH Brosens JJ. Recurrent pregnancy loss. Nat Rev Dis Prim. (2020) 6:98. doi: 10.1038/s41572-020-00228-z, PMID: 33303732

[7] Berkane N Verstraete L Uzan S. Recurrent miscarriage. Rev Prat. (2003) 53:1906–12., PMID: 14722979

[8] Tardito S Isella C Medico E Marchio L Bevilacqua E Hatzoglou M. The thioxotriazole copper(II) complex A0 induces endoplasmic reticulum stress and paraptotic death in human cancer cells. J Biol Chem. (2009) 284:24306–19. doi: 10.1074/jbc.M109.026583, PMID: 19561079 PMC2782024

[9] Shubin AV Demidyuk IV Komissarov AA Rafieva LM Kostrov SV. Cytoplasmic vacuolization in cell death and survival. Oncotarget. (2016) 7:55863–89. doi: 10.18632/oncotarget.10150, PMID: 27331412 PMC5342458

[10] Kim E Lee DM Seo MJ Lee HJ Choi KS. Intracellular ca(2 +) imbalance critically contributes to paraptosis. Front Cell Dev Biol. (2020) 8:607844. doi: 10.3389/fcell.2020.607844, PMID: 33585447 PMC7873879

[11] Zhuo Y Song Y. Prognostic and immunological implications of paraptosis-related genes in lung adenocarcinoma: Comprehensive analysis and functional verification of hub gene. Environ Toxicol. (2024) 40:396–411. doi: 10.1002/tox.24185, PMID: 38445368

[12] Liu C Wang L Sun Y Zhao X Chen T Su X. Probe synthesis reveals eukaryotic translation elongation factor 1 alpha 1 as the anti-pancreatic cancer target of BE-43547A(2). Angew Chem Int Ed Engl. (2022) 61:e202206953. doi: 10.1002/anie.202206953, PMID: 35705783

[13] Kunst C Tumen D Ernst M Tews HC Muller M Gulow K. Paraptosis-A distinct pathway to cell death. Int J Mol Sci. (2024) 25:11478. doi: 10.3390/ijms252111478, PMID: 39519031 PMC11546839

[14] Lee NP Chan CM Tung LN Wang HK Law S. Tumor xenograft animal models for esophageal squamous cell carcinoma. J BioMed Sci. (2018) 25:66. doi: 10.1186/s12929-018-0468-7, PMID: 30157855 PMC6116446

[15] Yamaguchi K Yokoi K Umezawa M Tsuchiya K Yamada Y Aoki S. Design, synthesis, and anticancer activity of triptycene-peptide hybrids that induce paraptotic cell death in cancer cells. Bioconjug Chem. (2022) 33:691–717. doi: 10.1021/acs.bioconjchem.2c00076, PMID: 35404581

[16] Hanson S Dharan A P VJ Pal S BG N Kar R. Paraptosis: a unique cell death mode for targeting cancer. Front Pharmacol. (2023) 14:1159409. doi: 10.3389/fphar.2023.1159409, PMID: 37397502 PMC10308048

[17] Grasso E Gori S Soczewski E Fernandez L Gallino L Vota D. Impact of the Reticular Stress and Unfolded Protein Response on the inflammatory response in endometrial stromal cells. Sci Rep. (2018) 8:12274. doi: 10.1038/s41598-018-29779-8, PMID: 30116009 PMC6095878

[18] Ishii T Yasuda K Miyazawa M Mitsushita J Johnson TE Hartman PS. Infertility and recurrent miscarriage with complex II deficiency-dependent mitochondrial oxidative stress in animal models. Mech Ageing Dev. (2016) 155:22–35. doi: 10.1016/j.mad.2016.02.013, PMID: 26944226

[19] Ramalho-Santos J Varum S Amaral S Mota PC Sousa AP Amaral A. Mitochondrial functionality in reproduction: from gonads and gametes to embryos and embryonic stem cells. Hum Reprod Update. (2009) 15:553–72. doi: 10.1093/humupd/dmp016, PMID: 19414527

[20] Capatina N Hemberger M Burton GJ Watson ED Yung HW. Excessive endoplasmic reticulum stress drives aberrant mouse trophoblast differentiation and placental development leading to pregnancy loss. J Physiol. (2021) 599:4153–81. doi: 10.1113/JP281994, PMID: 34269420

[21] Qin H Abulaiti A Maimaiti A Abulaiti Z Fan G Aili Y. Integrated machine learning survival framework develops a prognostic model based on inter-crosstalk definition of mitochondrial function and cell death patterns in a large multicenter cohort for lower-grade glioma. J Transl Med. (2023) 21:588. doi: 10.1186/s12967-023-04468-x, PMID: 37660060 PMC10474752

[22] Ritchie ME Phipson B Wu D Hu Y Law CW Shi W. limma powers differential expression analyses for RNA-sequencing and microarray studies. Nucleic Acids Res. (2015) 43:e47. doi: 10.1093/nar/gkv007, PMID: 25605792 PMC4402510

[23] Gustavsson EK Zhang D Reynolds RH Garcia-Ruiz S Ryten M. ggtranscript: an R package for the visualization and interpretation of transcript isoforms using ggplot2. Bioinformatics. (2022) 38:3844–6. doi: 10.1093/bioinformatics/btac409, PMID: 35751589 PMC9344834

[24] Gu Z Eils R Schlesner M. Complex heatmaps reveal patterns and correlations in multidimensional genomic data. Bioinformatics. (2016) 32:2847–9. doi: 10.1093/bioinformatics/btw313, PMID: 27207943

[25] Langfelder P Horvath S. WGCNA: an R package for weighted correlation network analysis. BMC Bioinf. (2008) 9:559. doi: 10.1186/1471-2105-9-559, PMID: 19114008 PMC2631488

[26] Hanzelmann S Castelo R Guinney J. GSVA: gene set variation analysis for microarray and RNA-seq data. BMC Bioinf. (2013) 14:7. doi: 10.1186/1471-2105-14-7, PMID: 23323831 PMC3618321

[27] Zheng Y Gao W Zhang Q Cheng X Liu Y Qi Z. Ferroptosis and autophagy-related genes in the pathogenesis of ischemic cardiomyopathy. Front Cardiovasc Med. (2022) 9:906753. doi: 10.3389/fcvm.2022.906753, PMID: 35845045 PMC9279674

[28] Wu T Hu E Xu S Chen M Guo P Dai Z. clusterProfiler 4. 0: A Univ Enrich tool interpreting Omics data. Innovation (Camb). (2021) 2:100141. doi: 10.1016/j.xinn.2021.100141, PMID: 34557778 PMC8454663

[29] Szczesny B Boorgula MP Chavan S Campbell M Johnson RK Kammers K. Multi-omics in nasal epithelium reveals three axes of dysregulation for asthma risk in the African Diaspora populations. Nat Commun. (2024) 15:4546. doi: 10.1038/s41467-024-48507-7, PMID: 38806494 PMC11133339

[30] Zhu R Yu X Li Y. Identification and validation of biomarkers associated with glycolysis in polycystic ovarian syndrome. Sci Rep. (2025) 15:27199. doi: 10.1038/s41598-025-11591-w, PMID: 40715316 PMC12297368

[31] Xie Q Zhang X Liu F Luo J Liu C Zhang Z. Identification and verification of immune-related genes for diagnosing the progression of atherosclerosis and metabolic syndrome. BMC Cardiovasc Disord. (2024) 24:405. doi: 10.1186/s12872-024-04026-3, PMID: 39095691 PMC11295872

[32] Liu P Xu H Shi Y Deng L Chen X. Potential molecular mechanisms of plantain in the treatment of gout and hyperuricemia based on network pharmacology. Evid Based Comp Alternat Med. (2020) 2020:3023127. doi: 10.1155/2020/3023127, PMID: 33149752 PMC7603577

[33] Robin X Turck N Hainard A Tiberti N Lisacek F Sanchez JC. pROC: an open-source package for R and S+ to analyze and compare ROC curves. BMC Bioinf. (2011) 12:77. doi: 10.1186/1471-2105-12-77, PMID: 21414208 PMC3068975

[34] Sachs MC. plotROC: A tool for plotting ROC curves. J Stat Softw. (2017) 79:2. doi: 10.18637/jss.v079.c02, PMID: 30686944 PMC6347406

[35] Orifjon S Jammatov J Sousa C Barros R Vasconcelos O Rodrigues P. Translation and adaptation of the adult developmental coordination disorder/dyspraxia checklist (ADC) into asian Uzbekistan. Sports (Basel). (2023) 11:135. doi: 10.3390/sports11070135, PMID: 37505622 PMC10383954

[36] Zhang X Chao P Zhang L Xu L Cui X Wang S. Single-cell RNA and transcriptome sequencing profiles identify immune-associated key genes in the development of diabetic kidney disease. Front Immunol. (2023) 14:1030198. doi: 10.3389/fimmu.2023.1030198, PMID: 37063851 PMC10091903

[37] Aran D Hu Z Butte AJ. xCell: digitally portraying the tissue cellular heterogeneity landscape. Genome Biol. (2017) 18:220. doi: 10.1186/s13059-017-1349-1, PMID: 29141660 PMC5688663

[38] Aran D. Cell-type enrichment analysis of bulk transcriptomes using xCell. Methods Mol Biol. (2020) 2120:263–76. doi: 10.1007/978-1-0716-0327-7_19, PMID: 32124326

[39] Ru Y Kechris KJ Tabakoff B Hoffman P Radcliffe RA Bowler R. The multiMiR R package and database: integration of microRNA-target interactions along with their disease and drug associations. Nucleic Acids Res. (2014) 42:e133. doi: 10.1093/nar/gku631, PMID: 25063298 PMC4176155

[40] Hao Y Hao S Andersen-Nissen E Mauck WM 3rd Zheng S Butler A. Integrated analysis of multimodal single-cell data. Cell. (2021) 184:3573–87 e29. doi: 10.1016/j.cell.2021.04.048, PMID: 34062119 PMC8238499

[41] Wei P Dong M Bi Y Chen S Huang W Li T. Identification and validation of a signature based on macrophage cell marker genes to predict recurrent miscarriage by integrated analysis of single-cell and bulk RNA-sequencing. Front Immunol. (2022) 13:1053819. doi: 10.3389/fimmu.2022.1053819, PMID: 36439123 PMC9692009

[42] Griss J Viteri G Sidiropoulos K Nguyen V Fabregat A Hermjakob H. ReactomeGSA - efficient multi-omics comparative pathway analysis. Mol Cell Proteomics. (2020) 19:2115–25. doi: 10.1074/mcp.TIR120.002155, PMID: 32907876 PMC7710148

[43] Jin S Guerrero-Juarez CF Zhang L Chang I Ramos R Kuan CH. Inference and analysis of cell-cell communication using CellChat. Nat Commun. (2021) 12:1088. doi: 10.1038/s41467-021-21246-9, PMID: 33597522 PMC7889871

[44] Trapnell C Cacchiarelli D Grimsby J Pokharel P Li S Morse M. The dynamics and regulators of cell fate decisions are revealed by pseudotemporal ordering of single cells. Nat Biotechnol. (2014) 32:381–6. doi: 10.1038/nbt.2859, PMID: 24658644 PMC4122333

[45] Gangat N Tefferi A. Myeloproliferative neoplasms and pregnancy: Overview and practice recommendations. Am J Hematol. (2021) 96:354–66. doi: 10.1002/ajh.26067, PMID: 33296529

[46] Yang Y Qiu J Xu Q Fan Y Wang H Qian H. The loss of dNK1/2 and EVT1 cells at the maternal-fetal interface is associated with recurrent miscarriagedagger. Biol Reprod. (2025) 112:119–29. doi: 10.1093/biolre/ioae136, PMID: 39303127

[47] Patronia MM Potiris A Mavrogianni D Drakaki E Karampitsakos T Machairoudias P. The expression of microRNAs and their involvement in recurrent pregnancy loss. J Clin Med. (2024) 13:3361. doi: 10.37766/inplasy2024.4.0116, PMID: 38929888 PMC11203554

[48] Sugiura-Ogasawara M. Recurrent pregnancy loss and obesity. Best Pract Res Clin Obstet Gynaecol. (2015) 29:489–97. doi: 10.1016/j.bpobgyn.2014.12.001, PMID: 25589398

[49] Kim JY Lee DM Woo HG Kim KD Lee HJ Kwon YJ. RNAi screening-based identification of USP10 as a novel regulator of paraptosis. Sci Rep. (2019) 9:4909. doi: 10.1038/s41598-019-40982-z, PMID: 30894572 PMC6427038

[50] Rajawat J Mir H Alex T Bakshi S Begum R. Involvement of poly(ADP-ribose) polymerase in paraptotic cell death of D. Discoid Apopt. (2014) 19:90–101. doi: 10.1007/s10495-013-0920-9, PMID: 24129923

[51] Wang WB Feng LX Yue QX Wu WY Guan SH Jiang BH. Paraptosis accompanied by autophagy and apoptosis was induced by celastrol, a natural compound with influence on proteasome, ER stress and Hsp90. J Cell Physiol. (2012) 227:2196–206. doi: 10.1002/jcp.22956, PMID: 21866552

[52] Wang C Zha YL Wang H Sun B Qiang WG Yuan Y. Carfilzomib promotes Iodine-125 seed radiation-induced apoptosis, paraptosis, and ferroptosis in esophageal squamous cell carcinoma by aggravating endoplasmic reticulum stress. Transl Oncol. (2025) 57:102393. doi: 10.1016/j.tranon.2025.102393, PMID: 40315760 PMC12098164

[53] Chen Y Kokic G Dienemann C Dybkov O Urlaub H Cramer P. Structure of the transcribing RNA polymerase II-Elongin complex. Nat Struct Mol Biol. (2023) 30:1925–35. doi: 10.1038/s41594-023-01138-w, PMID: 37932450 PMC10716050

[54] Bao Y Zhao TL Zhang ZQ Liang XL Wang ZX Xiong Y. High eukaryotic translation elongation factor 1 alpha 1 expression promotes proliferation and predicts poor prognosis in clear cell renal cell carcinoma. Neoplasma. (2020) 67:78–84. doi: 10.4149/neo_2019_190224N158, PMID: 31777262

[55] Tian L Gong L Hao C Feng Y Yao S Fei B. ELOA promotes tumor growth and metastasis by activating RBP1 in gastric cancer. Cancer Med. (2023) 12:18946–59. doi: 10.1002/cam4.6516, PMID: 37694492 PMC10557880

[56] Kawauchi J Inoue M Fukuda M Uchida Y Yasukawa T Conaway RC. Transcriptional properties of mammalian elongin A and its role in stress response. J Biol Chem. (2013) 288:24302–15. doi: 10.1074/jbc.M113.496703, PMID: 23828199 PMC3750133

[57] Miyata K Yasukawa T Fukuda M Takeuchi T Yamazaki K Sakumi K. Induction of apoptosis and cellular senescence in mice lacking transcription elongation factor, Elongin A. Cell Death Differ. (2007) 14:716–26. doi: 10.1038/sj.cdd.4402067, PMID: 17170753

[58] DeGregori J Johnson DG. Distinct and overlapping roles for E2F family members in transcription, proliferation and apoptosis. Curr Mol Med. (2006) 6:739–48. doi: 10.2174/1566524010606070739, PMID: 17100600

[59] Stevens C La Thangue NB. E2F and cell cycle control: a double-edged sword. Arch Biochem Biophys. (2003) 412:157–69. doi: 10.1016/S0003-9861(03)00054-7, PMID: 12667479

[60] Liu Z Wang C Tang Y Zhang X Pei J Liu H. ENO1 promotes trophoblast invasion regulated by E2F8 in recurrent miscarriage. FASEB J. (2024) 38:e23631. doi: 10.1096/fj.202302032RR, PMID: 38661062

[61] Krempler A Deckbar D Jeggo PA Lobrich M. An imperfect G2M checkpoint contributes to chromosome instability following irradiation of S and G2 phase cells. Cell Cycle. (2007) 6:1682–6. doi: 10.4161/cc.6.14.4480, PMID: 17637566

[62] Oshi M Newman S Tokumaru Y Yan L Matsuyama R Endo I. High G2M pathway score pancreatic cancer is associated with worse survival, particularly after margin-positive (R1 or R2) resection. Cancers (Basel). (2020) 12:2871. doi: 10.3390/cancers12102871, PMID: 33036243 PMC7599494

[63] Oshi M Patel A Le L Tokumaru Y Yan L Matsuyama R. G2M checkpoint pathway alone is associated with drug response and survival among cell proliferation-related pathways in pancreatic cancer. Am J Cancer Res. (2021) 11:3070–84., PMID: 34249445 PMC8263638

[64] Jing MY Xie LD Chen X Zhou Y Jin MM He WH. Circ-CCNB1 Modulates Trophoblast Proliferation and Invasion in Spontaneous Abortion by Regulating miR-223/SIAH1 axis. Endocrinology. (2022) 163:bqac093. doi: 10.1210/endocr/bqac093, PMID: 35731831 PMC9290912

[65] Meng N Wang X Shi Y Mao Y Yang Q Ju B. miR-3074-5p/CLN8 pathway regulates decidualization in recurrent miscarriage. Reproduction. (2021) 162:33–45. doi: 10.1530/REP-21-0032, PMID: 34044364

[66] Oshi M Takahashi H Tokumaru Y Yan L Rashid OM Matsuyama R. G2M cell cycle pathway score as a prognostic biomarker of metastasis in estrogen receptor (ER)-positive breast cancer. Int J Mol Sci. (2020) 21:2921. doi: 10.3390/ijms21082921, PMID: 32331421 PMC7215898

[67] Tian J Jiang L Li H Dan J Luo Y. The dual role of the DREAM/G2M pathway in non-tumorigenic immortalization of senescent cells. FEBS Open Bio. (2024) 14:331–43. doi: 10.1002/2211-5463.13748, PMID: 38073074 PMC10839291

[68] AbuliHaiti Z Li W Yang L Zhang H Du A Tang N. Hypoxia-driven lncRNA CTD-2510F5.4: a potential player in hepatocellular carcinoma’s prognostic stratification, cellular behavior, tumor microenvironment, and therapeutic response. Mol Biol Rep. (2024) 51:905. doi: 10.1007/s11033-024-09826-6, PMID: 39133347

[69] Fellbrich G Romanski A Varet A Blume B Brunner F Engelhardt S. NPP1, a Phytophthora-associated trigger of plant defense in parsley and Arabidopsis. Plant J. (2002) 32:375–90. doi: 10.1046/j.1365-313X.2002.01454.x, PMID: 12410815

[70] Jones JDG Staskawicz BJ Dangl JL. The plant immune system: From discovery to deployment. Cell. (2024) 187:2095–116. doi: 10.1016/j.cell.2024.03.045, PMID: 38670067

[71] Duxbury Z Wang S MacKenzie CI Tenthorey JL Zhang X Huh SU. Induced proximity of a TIR signaling domain on a plant-mammalian NLR chimera activates defense in plants. Proc Natl Acad Sci U S A. (2020) 117:18832–9. doi: 10.1073/pnas.2001185117, PMID: 32709746 PMC7414095

[72] Hu Z Chai J. Structural mechanisms in NLR inflammasome assembly and signaling. Curr Top Microbiol Immunol. (2016) 397:23–42. doi: 10.1007/978-3-319-41171-2_2, PMID: 27460803

[73] Zhou Y Hu L Jiang L Liu S. Genome-wide identification, characterization, and transcriptional analysis of the metacaspase gene family in cucumber (Cucumis sativus). Genome. (2018) 61:187–94. doi: 10.1139/gen-2017-0174, PMID: 29461868

[74] Ding J Zhang Y Cai X Zhang Y Yan S Wang J. Extracellular vesicles derived from M1 macrophages deliver miR-146a-5p and miR-146b-5p to suppress trophoblast migration and invasion by targeting TRAF6 in recurrent spontaneous abortion. Theranostics. (2021) 11:5813–30. doi: 10.7150/thno.58731, PMID: 33897883 PMC8058722

[75] Kanki K Ii M Terai Y Ohmichi M Asahi M. Bone marrow-derived endothelial progenitor cells reduce recurrent miscarriage in gestation. Cell Transplant. (2016) 25:2187–97. doi: 10.3727/096368916X692753, PMID: 27513361

[76] Liu S Wei H Li Y Huang C Lian R Xu J. Downregulation of ILT4(+) dendritic cells in recurrent miscarriage and recurrent implantation failure. Am J Reprod Immunol. (2018) 80:e12998. doi: 10.1111/aji.12998, PMID: 29904967

[77] Qian ZD Huang LL Zhu XM. An immunohistochemical study of CD83- and CD1a-positive dendritic cells in the decidua of women with recurrent spontaneous abortion. Eur J Med Res. (2015) 20:2. doi: 10.1186/s40001-014-0076-2, PMID: 25563385 PMC4301856

[78] Zhao QY Li QH Fu YY Ren CE Jiang AF Meng YH. Decidual macrophages in recurrent spontaneous abortion. Front Immunol. (2022) 13:994888. doi: 10.3389/fimmu.2022.994888, PMID: 36569856 PMC9781943

[79] Alshahrani MY Saleh RO Hjazi A Bansal P Kaur H Deorari M. Molecular mechanisms of tumorgenesis and metastasis of long non-coding RNA (lncRNA) NEAT1 in human solid tumors; an update. Cell Biochem Biophys. (2024) 82:593–607. doi: 10.1007/s12013-024-01287-9, PMID: 38750383

[80] Li L Li G Guan R Ma H Xing Q. Inhibition of long non-coding RNA NEAT1 suppressed the epithelial mesenchymal transition through the miR-204-5p/Six1 axis in asthma. PloS One. (2024) 19:e0312020. doi: 10.1371/journal.pone.0312020, PMID: 39423195 PMC11488729

[81] Teng L Liu P Song X Wang H Sun J Yin Z. Long non-coding RNA nuclear-enriched abundant transcript 1 (NEAT1) represses proliferation of trophoblast cells in rats with preeclampsia via the microRNA-373/FLT1 axis. Med Sci Monit. (2020) 26:e927305. doi: 10.12659/MSM.927305, PMID: 33093438 PMC7590520

[82] Jiang X Guo S Zhang Y Zhao Y Li X Jia Y. LncRNA NEAT1 promotes docetaxel resistance in prostate cancer by regulating ACSL4 via sponging miR-34a-5p and miR-204-5p. Cell Signal. (2020) 65:109422. doi: 10.1016/j.cellsig.2019.109422, PMID: 31672604

[83] Yagel S. The developmental role of natural killer cells at the fetal-maternal interface. Am J Obstet Gynecol. (2009) 201:344–50. doi: 10.1016/j.ajog.2009.02.030, PMID: 19788966

[84] James-Allan LB Buckley RJ Whitley GS Cartwright JE. The phenotype of decidual CD56+ lymphocytes is influenced by secreted factors from decidual stromal cells but not macrophages in the first trimester of pregnancy. J Reprod Immunol. (2020) 138:103082. doi: 10.1016/j.jri.2020.103082, PMID: 31982613

[85] Zhang Y Wang Y Wang XH Zhou WJ Jin LP Li MQ. Crosstalk between human endometrial stromal cells and decidual NK cells promotes decidualization *in vitro* by upregulating IL−25. Mol Med Rep. (2018) 17:2869–78. doi: 10.3892/mmr.2017.8267, PMID: 29257317 PMC5783502

[86] Fonseca BM Cunha SC Goncalves D Mendes A Braga J Correia-da-Silva G. Decidual NK cell-derived conditioned medium from miscarriages affects endometrial stromal cell decidualisation: endocannabinoid anandamide and tumour necrosis factor-alpha crosstalk. Hum Reprod. (2020) 35:265–74. doi: 10.1093/humrep/dez260, PMID: 31990346

[87] Zhu J Song G Zhou X Han TL Yu X Chen H. CD39/CD73 dysregulation of adenosine metabolism increases decidual natural killer cell cytotoxicity: implications in unexplained recurrent spontaneous abortion. Front Immunol. (2022) 13:813218. doi: 10.3389/fimmu.2022.813218, PMID: 35222389 PMC8866181

[88] Mani S Garifallou J Kim SJ Simoni MK Huh DD Gordon SM. Uterine macrophages and NK cells exhibit population and gene-level changes after implantation but maintain pro-invasive properties. Front Immunol. (2024) 15:1364036. doi: 10.3389/fimmu.2024.1364036, PMID: 38566989 PMC10985329

[89] Patel U Rajasingh S Samanta S Cao T Dawn B Rajasingh J. Macrophage polarization in response to epigenetic modifiers during infection and inflammation. Drug Discov Today. (2017) 22:186–93. doi: 10.1016/j.drudis.2016.08.006, PMID: 27554801 PMC5226865

[90] Zhao X Jiang Y Luo S Zhao Y Zhao H. Intercellular communication involving macrophages at the maternal-fetal interface may be a pivotal mechanism of URSA: a novel discovery from transcriptomic data. Front Endocrinol (Lausanne). (2023) 14:973930. doi: 10.3389/fendo.2023.973930, PMID: 37265689 PMC10231036

[91] Parvanov D Ganeva R Arsov K Decheva I Handzhiyska M Ruseva M. Association between endometrial senescent cells and immune cells in women with repeated implantation failure. J Assist Reprod Genet. (2023) 40:1631–8. doi: 10.1007/s10815-023-02821-z, PMID: 37145373 PMC10352182

[92] Tidball JG Flores I Welc SS Wehling-Henricks M Ochi E. Aging of the immune system and impaired muscle regeneration: A failure of immunomodulation of adult myogenesis. Exp Gerontol. (2021) 145:111200. doi: 10.1016/j.exger.2020.111200, PMID: 33359378 PMC7855614

[93] Bean-Knudsen DE Wagner JE. The wire-bar cage top as a barrier to breeding and genetic contamination of laboratory mice. Lab Anim Sci. (1987) 37:350–1., PMID: 3613517

[94] Zhang J Dunk CE Kwan M Jones RL Harris LK Keating S. Human dNK cell function is differentially regulated by extrinsic cellular engagement and intrinsic activating receptors in first and second trimester pregnancy. Cell Mol Immunol. (2017) 14:203–13. doi: 10.1038/cmi.2015.66, PMID: 26277900 PMC5301153

