# Supplementary figures and images for: Identification and validation of paraptosis-related biomarkers in recurrent miscarriage
Source: Front Immunol. 2025 Nov 5;16:1656650. doi: 10.3389/fimmu.2025.1656650 (PMC12626843; doi:10.3389/fimmu.2025.1656650)

### Correlation vs Soft Threshold

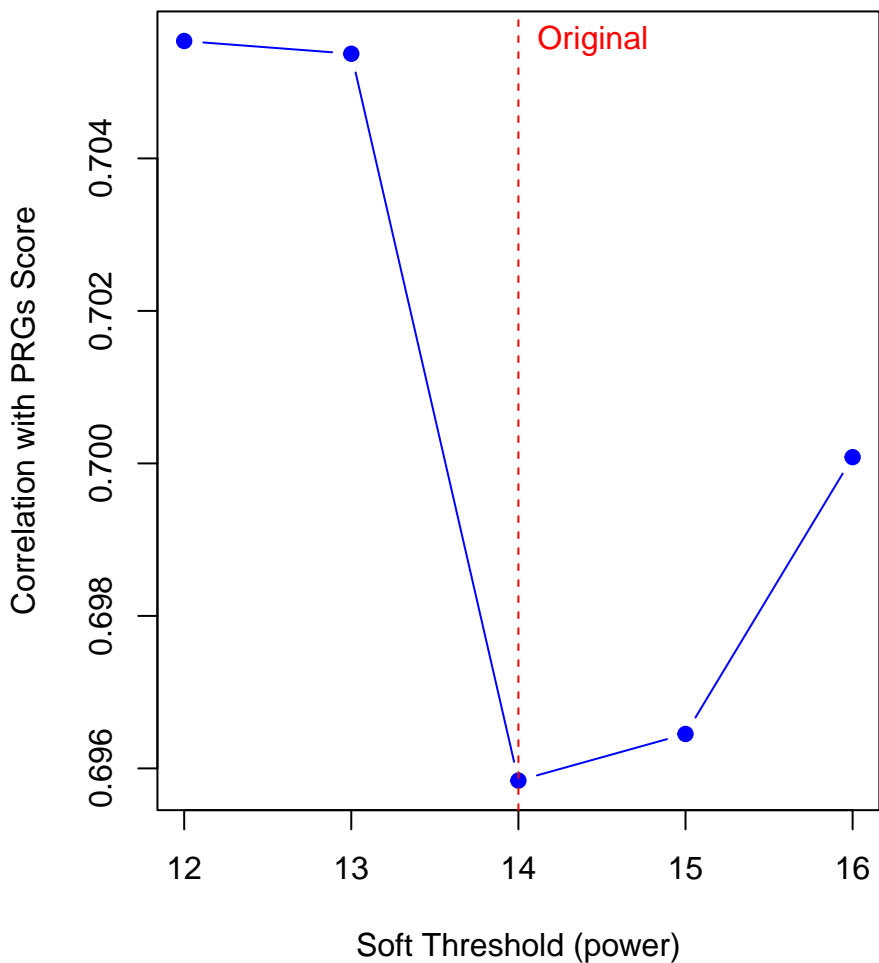

### Significance vs Soft Threshold

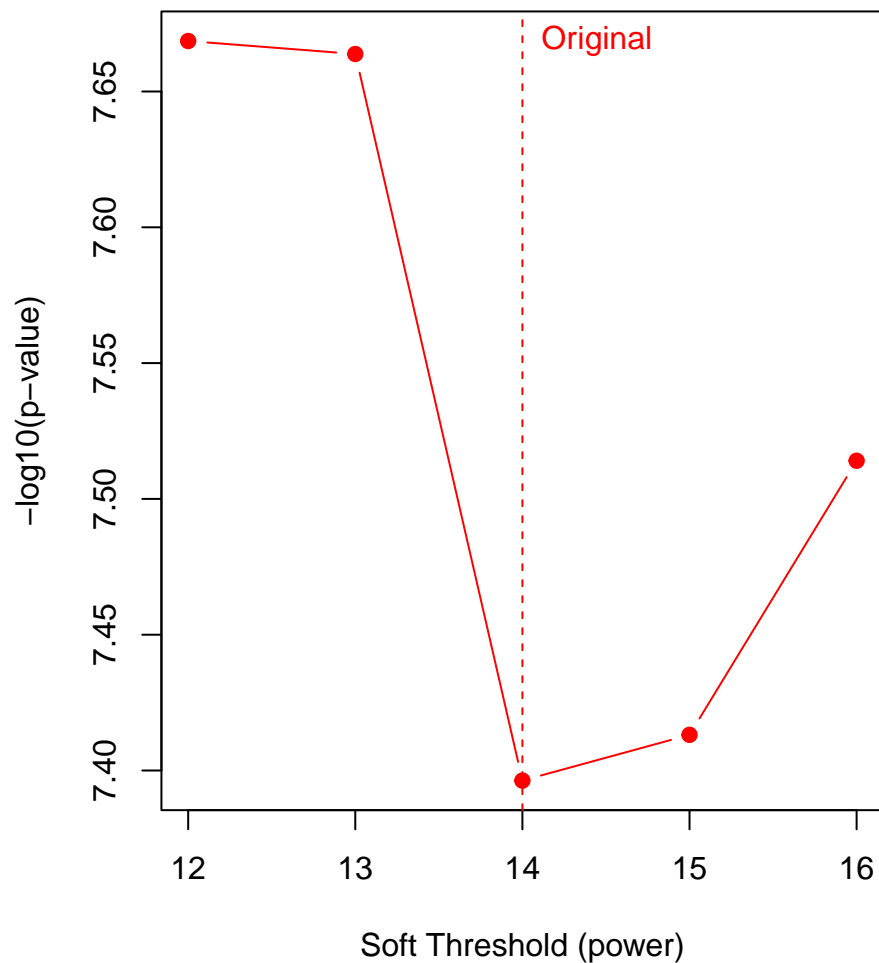

Supplement: Supplementary file 1 [file DataSheet1.pdf]

# Jaccard Similarity between Module Sizes

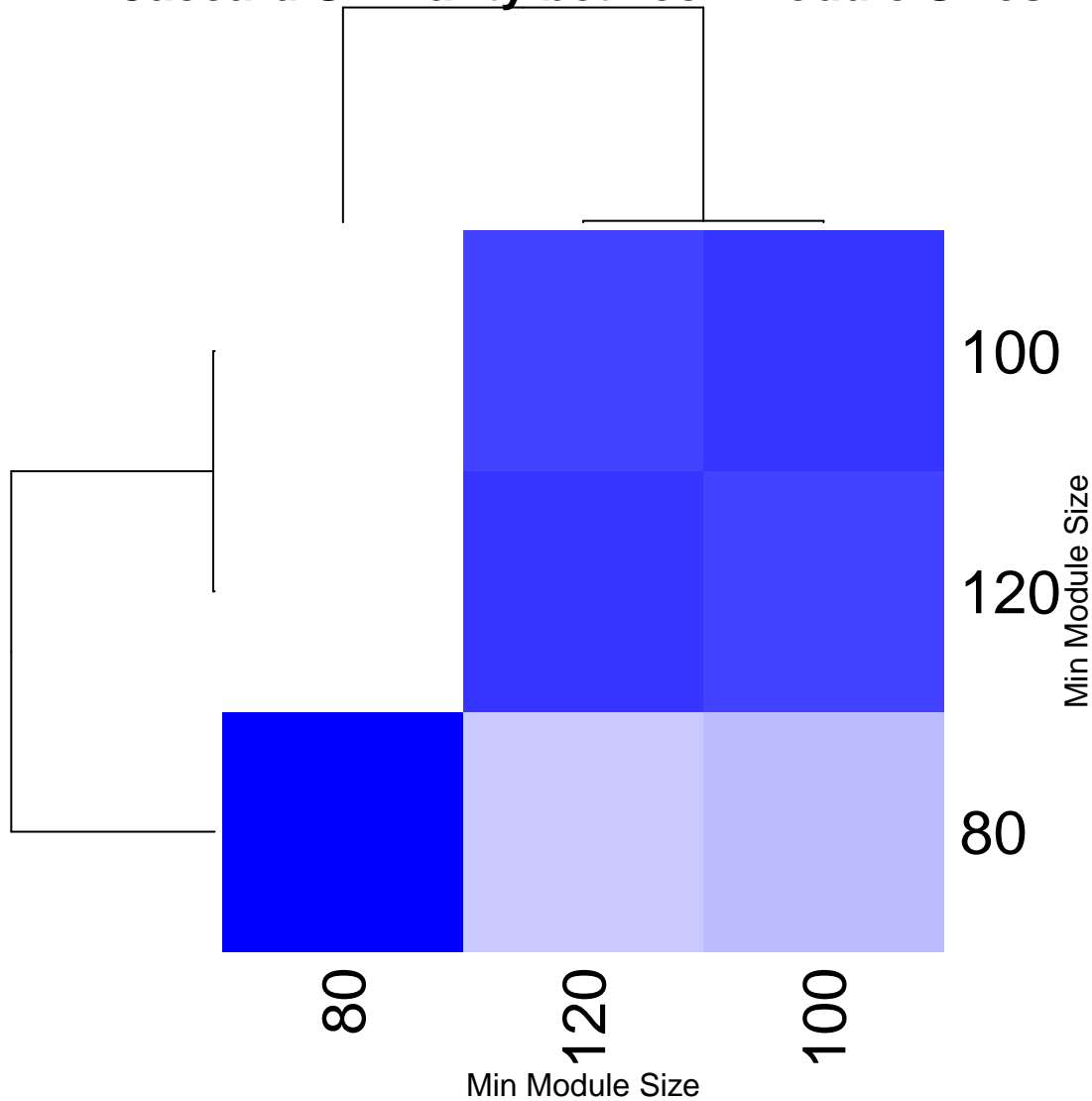

Supplement: Supplementary file 2 [file DataSheet2.pdf]

### Mean Connectivity vs Power

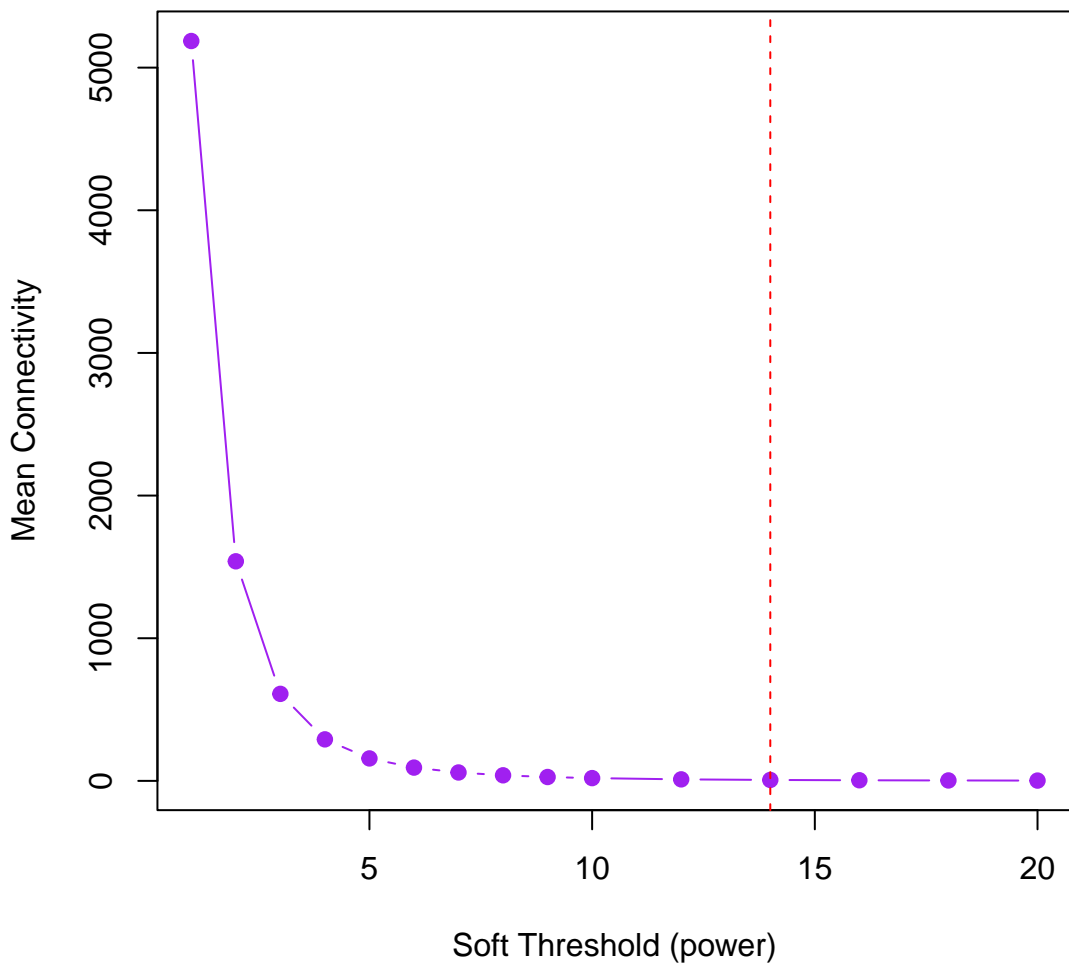

### Median Connectivity vs Power

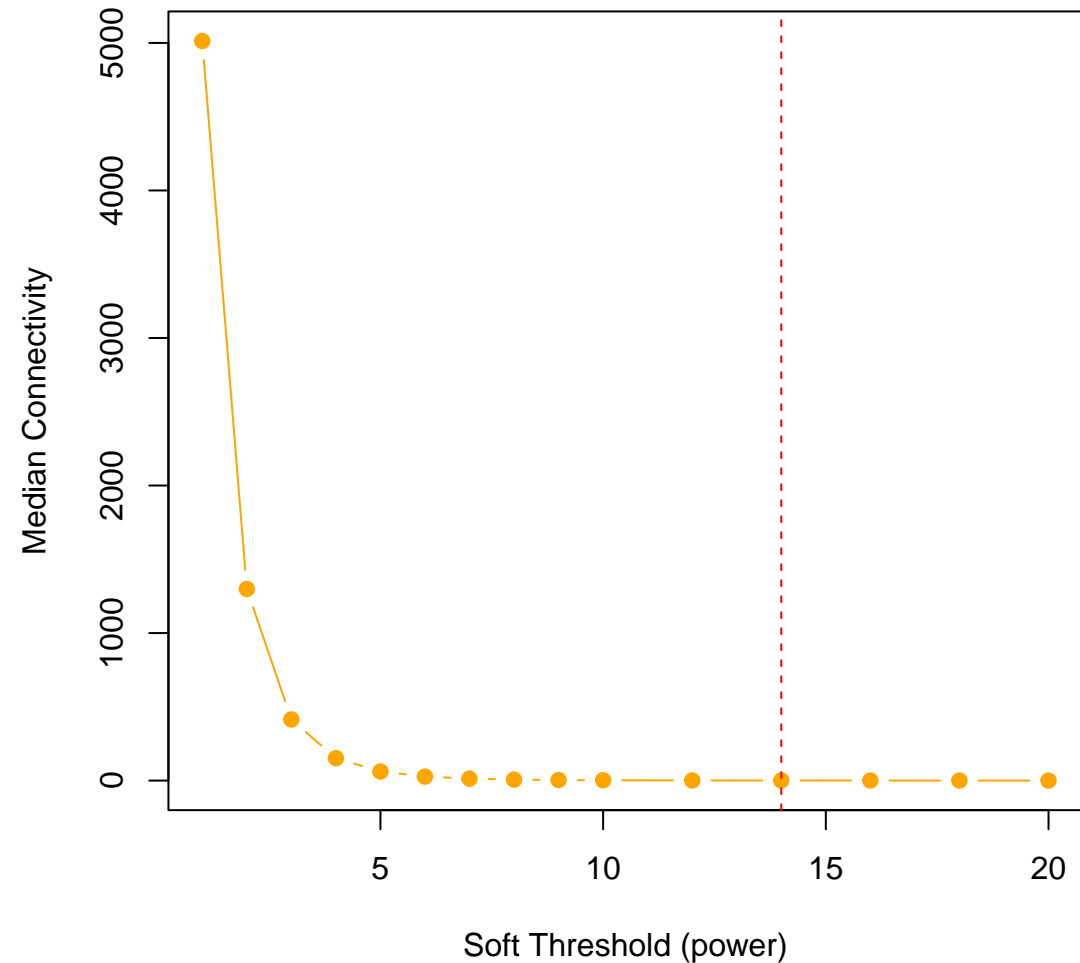

Supplement: Supplementary file 3 [file DataSheet3.pdf]
