# Supplementary material for: Identification and validation of paraptosis-related biomarkers in recurrent miscarriage
Source: Front Immunol. 2025 Nov 5;16:1656650. doi: 10.3389/fimmu.2025.1656650 (PMC12626843; doi:10.3389/fimmu.2025.1656650)
Supplement: Supplementary file 4 [file Table1.docx]

Supplementary Material

# Supplementary Tables

**Tables S1:a list of 66 PRGs**

| \| gene \| \| --- \| \| CAMK2B \| \| PRKACG \| \| MARK4 \| \| SSTR5 \| \| TAAR5 \| \| USP10 \| \| PRKAG3 \| \| HACD2 \| \| NT5C \| \| INSRR \| \| SSTR3 \| \| TAAR9 \| \| HSPB8 \| \| PLPP2 \| \| G6PC2 \| \| GUCY2EP \| \| CDK4 \| \| RGR \| \| ADGRG1 \| \| UOCRCI \| \| TNK2 \| \| RNF181 \| \| MKNK2 \| \| UBE2U \| \| MYLK \| \| CTDSP2 \| \| LCK \| \| GPR15 \| \| ATP23 \| \| LPAR1 \| \| PI4KB \| \| DSTYK \| \| CFD \| \| PPP3CA \| \| CCR4 \| \| PRAG1 \| \| CDKN3 \| \| GPR153 \| \| DDIT3 \| \| MAPK8 \| \| MAP2K2 \| \| MAPK1 \| \| MAPK14 \| \| IGFIR \| \| PDCD6IP \| \| CASP9 \| \| ERN1 \| \| ATF6 \| \| XBP1 \| \| AKT1 \| \| EIF2S1 \| \| HSPA5 \| \| CASP4 \| \| CASP3 \| \| CASP7 \| \| ITPR3 \| \| RYR1 \| \| RYR2 \| \| MCU \| \| TNFRSF19 \| \| PDCD5 \| \| CSF1 \| \| TP3 \| \| NFKB1 \| \| PEBP1 \| \| PHB \| |
| --- | --- | --- | --- | --- | --- | --- | --- | --- | --- | --- | --- | --- | --- | --- | --- | --- | --- | --- | --- | --- | --- | --- | --- | --- | --- | --- | --- | --- | --- | --- | --- | --- | --- | --- | --- | --- | --- | --- | --- | --- | --- | --- | --- | --- | --- | --- | --- | --- | --- | --- | --- | --- | --- | --- | --- | --- | --- | --- | --- | --- | --- | --- | --- | --- | --- | --- | --- |
